# Combining Different Natural Plant Extracts to Stabilize the Antioxidative Activity of Dragon’s Blood

**DOI:** 10.3390/life14070786

**Published:** 2024-06-21

**Authors:** Ying-Zhen Su, Pei-Luen Lu

**Affiliations:** Department of Life Sciences, National Taitung University, Taitung 950309, Taiwan; janice8605@gmail.com

**Keywords:** *Hibiscus sabdariffa*, *Clitoria ternatea*, *Hylocereus* sp., *Pandanus amaryllifolius*, *Camellia sinensis*, dragon’s blood, *Dracaena cochinchinensis*, *Daemonorops draco*, antioxidant activity

## Abstract

Dragon’s blood (血竭) is a traditional Chinese medicine known for its wound hemostasis, blood circulation, and stasis properties. Recently, it has also been utilized in cosmetics, though its antioxidant capacity remains unclear. This study aims to stabilize the bioactivity of dragon’s blood using various plant extracts. We evaluated single plant extracts and their combinations to identify the conditions that maintained the antioxidant capacity of dragon’s blood the longest. Selected plants included *Hibiscus sabdariffa*, *Clitoria ternatea*, *Hylocereus* sp., *Pandanus amaryllifolius*, and *Camellia sinensis*. We used two sources of dragon’s blood: *Daemonorops draco* and *Dracaena cochinchinensis*. Extraction conditions were optimized and antioxidant activity was assessed using the free radical scavenging ability of 1,1-diphenyl-2-picrylhydrazyl (DPPH), total anthocyanin concentration (TAC), total polyphenol content (TPC), the free radical scavenging activity of ABTS, and a ferric reducing antioxidant power (FRAP) assay. The results showed that all plant extracts exhibited high antioxidant capacity. *Clitoria ternatea* had the highest DPPH scavenging ability at 93.81%, with the best combination being green tea and *Daemonorops draco* at 92.57%. *Clitoria ternatea* had the highest TPC at 9921 mg GAE/100 g, with the best combination (green tea and *Dracaena cochinchinensis*) at 10500 mg GAE/100 g. ABTS activity was highest for green tea at 98.3%, with the best combination (*Clitoria ternatea* and *Daemonorops draco*) at 93.29%. The FRAP assay showed that green tea had the highest electron-donating potential at 3.85 mg/mL, with the best combination (*Daemonorops draco* and *Dracaena cochinchinensis*) at 3.71 mg/mL. This study advances our understanding of the antioxidant properties of these plants and the traditional Chinese medicine dragon’s blood, enhancing the efficacy of dragon’s blood in skincare and cosmetics. Moreover, the application of these extracts could rejuvenate local agriculture, impacting the skincare, cosmetics, and sustainable agriculture sectors.

## 1. Introduction

In ethnobotany, using dragon’s blood canincrease the healing popularity and clinical application when there is a wound [1,2], including antihemorrhagic [3], immunomodulatory [4], antioxidant [5], and active compounds in antimutagenic agents [6]. Chinese herbal medicines have a long history of use in traditional medicine and are still widely used today with high safety and low toxicity [7]. Several plant *Dracaena* species, also known as the main type of dragon’s blood, have been used as a Chinese medicinal material. They promote blood circulation, remove blood stasis, and reduce swelling and pain, and they can be used as an astringent, as well as for hemostasis [1,2]. Previous studies have confirmed that its extracts have ingredients that whiten, condition, and make skin smooth and firm. Dragon’s blood has been used as herbal medicine for over 1500 years, and its main biological activity is caused by several phenolic compounds [8,9]. It is derived from the red resin of dragon’s blood trees [10]. Dragon’s blood was documented to contain red resins extracted from six different plant genera, namely, *Dracaena*, *Daemonorops*, *Calamus*, *Pterocarpus*, *Eucalyptus*, and *Croton* [11,12,13]. Amazonians use dragon’s blood (fresh or dry powder) to treat sore throats, hives, cancer, vaginal fungal disease, mosquito bites, hemostasis, wound healing, and diarrhea [14,15,16,17,18]. There are many different resins available on the international market from different plant species called dragon’s blood [13]. Previous studies have shown that phenolic compounds may have comprehensive effects on neurorescue, neuroprotection, immune function, and antioxidant effects [19,20,21]. Additional in vitro studies testing for antioxidant properties have demonstrated the high scavenging capacity for hydroxyl radicals (•OH) and hydrogen peroxide (H_2_O_2_), two known agents of lipid peroxidation and cell membrane damage [22].

Roselle flowers (*Hibiscus sabdariffa* L.) have been used in food and beverages in many countries for a long time, such as in jellies, jams, sauces, and preserves [23,24,25]. The leaves or calyces are also traditionally used to prepare beverages, as they are rich in anthocyanins and have antioxidant properties and are used as diuretics and sedatives [26]. Anthocyanins are natural water-soluble pigments and one of the derivatives of flavonoids in phenolic compounds [25]. The high anthocyanin content in roselle petals is a good coloring agent and a potential source of antioxidants [27]. It also has properties associated with anti-diabetic, anti-inflammatory, and anti-cancer effects [28,29,30]. Among them, anthocyanins in roselles are related to antioxidant activity [31,32].

The butterfly pea flower (*Clitoria ternatea* L.) is a member of the legume family with a climbing stem. It is distributed in tropical Asia, the Philippine Islands, and Madagascar [33] and was introduced to Taiwan in 1920. Its roots have a strong bitter taste and have cooling, laxative, diuretic, anthelmintic, and anti-inflammatory properties, and they can cure severe bronchitis, asthma, and mania [34]. The butterfly pea flower extract was found to have anxiolytic, antidepressant, and antistress effects [35]. However, using its roots can cause a miscarriage among pregnant women, and its sticking can cause abdominal swelling, abnormal mucus, and fever [36,37]. The petals of butterfly pea flowers are rich in anthocyanins, which are useful natural colorants with great potential for use in various foods and beverages [38]. Hence, they are used as food colorants in Southeast Asia [39]. Butterfly pea flower extract not only imparts beautiful color to the food system but also has a wide range of pharmacological effects, promoting good health [40,41]. The beneficial effects of polyphenols in butterfly pea flower extract are related to their ability to scavenge free radicals [42,43,44]. Due to the medicinal properties of its extract, which features anti-inflammatory and analgesic properties, it has been used as a potential drug in recent years. More recently, it has been shown by studies to have anti-cancer properties [35,44].

Red dragon fruit (*Hylocereus* sp.) belongs to the Cactaceae family. Originating from Mexico, it was first introduced to Southeast Asia by the French in Vietnam at least 100 years ago, and its cultivation has spread to Thailand, Malaysia, and Indonesia [45]. There are several types of dragon fruit; among them, *Hylocereus undatus*, *Hylocereus costaricensis*, and *Hylocereus polyrhizus* are the most widely cultivated, with a red peel but white pulp, red fruit peel, and red pulp, respectively. They also have betaine pigments, with red-purple beta-betaine and orange-yellow betaine [46,47]. The leaves, roots, seeds, peels, oils, and pulps of these plants are rich in phenolic compounds that can act as hydrogen donors or free radical scavengers with antioxidant activity [48,49,50]. Red dragon fruit (*Hylocereus* sp.) is mainly eaten raw or in the form of jellies, juices, jams, and desserts [51,52].

The red dragon fruit flower is not only beautiful but also edible, delicious, and has health benefits. Fiber-rich pitaya peels have an antioxidant capacity for colorants and polyphenols, as analyzed by DPPH assays [53,54]. The composition of this fruit is rich in bioactive compounds such as beta-carotene, beta-anthocyanin, lycopene, vitamin E, vitamin C, and polyphenols [52,55,56,57].

Pandan leaves (*Pandanus amaryllifolius*), also known as Xianglintou, grow on tropical moist acidic soil and are known for their medicinal properties [58]. The ground planting conditions are relatively extensive, the light requirements are not high, and adaptability to the environment is strong. Pandan thrives in hillside slopes, courtyards, and farms [58]. The plant is native to Southeast Asia and thrives in tropical regions such as the Pacific, Africa, South Asia, and Australia [59]. It was introduced to Taiwan by Vietnam about 20 years ago. In Southeast Asia, medicinal leaves are used to rejuvenate the body, reduce fever, and relieve indigestion and flatulence [60]. The leaves contain essential oils, carotenoids, tocopherols and tocotrienols [7], quercetin [61], alkaloids [62], fatty acids [63], and nonspecific lipid transfer proteins [64].

Phenolic compounds in herbal medicines act as antioxidants due to their redox properties, allowing them to act as reducing agents, hydrogen donors, and metal chelators [59]. Pandan leaves are also a popular green food colorant due to their high chlorophyll content [65]. With a taro aroma, it is used as a seasoning in Indo-Chinese dishes [66]. Most of these herbs and spices are believed to be associated with high antioxidant activity and have numerous benefits for the human body [67]. Traditionally, this plant has also been considered a medicinal plant for treating gout, hyperglycemia, hypertension, and rheumatism [66]. In addition, medicinal plants have been proven to have antimicrobial, antioxidant, antiviral, hypoglycemic, and tumor growth inhibition properties [59,67,68,69,70,71].

Numerous factors influence extraction efficiency, including the extraction method, solvent type, concentration, plant material, extraction duration, and temperature [72]. Plants demonstrate varying antioxidant capacities and anthocyanin contents under different extraction conditions. Consequently, it is imperative to initially evaluate the extraction conditions and subsequently select analogous conditions for pairing. Thereafter, the paired compound extract should be utilized to ascertain the optimal conditions.

This research aims to support local agriculture by enhancing the added value of their products, thereby improving their income. Dragon’s blood, an important component of traditional Chinese medicine, is the focus of this study. The specific objectives of this study are as follows: 1. To determine which combination of natural plant extracts (from roselles, butterfly pea flowers, green tea, pandan leaves, and red dragon fruit flowers, both dry and wet) can extend the antioxidant activity of dragon’s blood. 2. To investigate whether these combinations of plant extracts can effectively maintain or enhance antioxidant activity. The significance of this study lies in its potential to provide valuable insights into the optimal extraction conditions for maximizing the efficacy of dragon’s blood. By identifying the best combinations of plant extracts to sustain or enhance the antioxidant properties, this research can contribute to the development of more effective medicinal products. Furthermore, this project is expected to contribute to local agricultural development by increasing the economic value of agricultural products, thus improving the income of local farmers.

## 2. Materials and Methods

### 2.1. Sample Collection

We utilized five different natural plant materials, excluding the chemically synthesized green tea extract. Most of these plant materials were sourced from local organic farmers: (1) The flowers of *Hibiscus sabdariffa* were purchased from the Organic Farmers’ Market, Taitung, Taiwan. (2) The flowers of *Clitoria ternatea* were purchased from the Organic Farmers’ Markets, Nantou, Taiwan (3) Chemical green tea extract was purchased from Chengyi Chemical Materials Co., Ltd., Taipei, Taiwan (4) *Clitoria ternatea* was purchased from the Organic Farmers’ markets, Taitung, Taiwan. (5) *Pandanus amaryllifolius* leaves were purchased from the Organic Farmers Market, Pingtung, Taiwan. (6) The flowers of Red dragon fruit (*Hylocereus* sp.) were purchased from the Organic FarmersMarket, Taitung, Taiwan. The materials for the traditional Chinese medicine dragon’s blood were sourced from various locations: (1) The Jinji brand was purchased from a Chinese medicine store in Dihua Street, Taipei, Taiwan. (2) The Jinxing brand was purchased from a Chinese medicine shop, in Dihua Street, Taipei, Taiwan. (3) The Baozhu brand was purchased from a Chinese medicine storein Dihua Street, Taipei, Taiwan. (4) The Crown brand was purchased from a Chinese medicine storein Dihua Street, Taipei, Taiwan. (5) The Xianfeng brand was purchased from a Chinese medicine store in Dihua Street, Taipei, Taiwan. (6) The Hong Kong orchid brand of dried dragon’s blood was purchased from a Chinese medicine storein Dihua Street, Taipei, Taiwan. (7) The Baozhu brand Qilin dragon’s blood was purchased from a Chinese medicine store in Taitung, Taiwan. (8) The Chungyaon Qilin dragon’s blood was purchased from a Chinese medicine storein Dihua Street, Taipei, Taiwan. (9) Indonesian dragon’s blood was purchased from Sumatra, Indonesia. (10) Sword leaf dragon’s blood (*Dracaena cochinchinensis*) was purchased from a Chinese medicine store, Guangzhou, China. (11) Yemen Socotra dragon’s blood (*Dracaena cinnabari*) was purchased from Socotra, Yemen. (12) The seeds of Draconis of the genus *Daemonorops* family Palmaceae (*Daemonorops draco*) were purchased from Sumatra, Indonesia.

### 2.2. Extraction Condition

The extraction conditions are given as follows. Plant samples were mixed with 0.1 g of powder and a 1 mL solution (30% ethanol, 70% ethanol, 30% methanol, 70% methanol, and ultrapure water), and the heating temperatures were 25 °C, 50 °C, and 75 °C, respectively. After extracting for 20 min, the centrifuge was placed to centrifuge at 6000 rpm for 15 min and the supernatant was taken. For dragon’s blood samples, 0.01 g of powder was mixed with 1 mL of solution and the subsequent steps were the same [73].

### 2.3. Analysis of DPPH Free Radical Scavenging Ability

The DPPH free radical scavenging capacity was analyzed according to the method described by Vulić et al. [72] with modifications. Specifically, 0.0394 g of DPPH was dissolved in 70% ethanol to prepare a 100 mL DPPH solution. A 0.01 mL aliquot of the sample was mixed with 0.19 mL of the DPPH solution and allowed to stand in the dark at room temperature for 10 min. The absorbance was measured at 517 nm using a spectrophotometer [72]. Vitamin C was used as a standard. The percentage of DPPH reduction was calculated using the following equation:

% DPPH reduction = (1 − As/Ac) × 100

Ac = absorbance of control;

As = absorbance of the sample.

### 2.4. Total Anthocyanin Concentration Analysis (TAC)

Total anthocyanin concentration was determined using the pH differential method as described by Vulić et al. [72,74]. Two buffer solutions were prepared: pH 1.0 (0.025 M KCl) and pH 4.5 (0.4 M CH_3_COONa). Samples were diluted to fall within a measurable absorbance range. A 0.1 mL aliquot of the sample was mixed with 0.9 mL of each buffer solution and allowed to stand for 1 h. Absorbance values were measured at 700 nm and 510 nm. The TAC values were calculated using the following equation:

Absorbance (A) = (A510 − A700) pH 1.0 − (A510 − A700) pH 4.5

TAC (mg/L) = (A × MW × DF × 1000)/(ε × l)

A = absorbance of control;

MW = 449.2 (molecular weight of anthocyanin-3-glucoside);

DF = 10 for natural plants (dilution factor), 100 for Dracaena;

ε = 26,900 (molar absorptivity);

l = path length (cm).

### 2.5. Analysis of Total Polyphenol Concentration (TPC)

The total polyphenol content was determined using the Folin–Ciocalteu method as modified by Vulić et al. [72,74]. A 10% sodium carbonate solution was prepared by dissolving 10 g of sodium carbonate in water to a final volume of 100 mL. A gallic acid solution was prepared by dissolving 0.05 g of gallic acid in water to a final volume of 100 mL (500 mg/L final concentration). A 0.01 mL aliquot of the sample was mixed with 0.6 mL of ultrapure water and 0.05 mL of Folin–Ciocalteu reagent and allowed to react for 8 min. Subsequently, 0.15 mL of a sodium carbonate solution was added, and the mixture was diluted to 1 mL with ultrapure water. After a 2-h reaction at room temperature, the absorbance was measured at 765 nm using a spectrophotometer. The total phenolic content was expressed in mg/L gallic acid equivalents (GAE). A mixture of water and reagent was used as a blank. All analyses were performed in triplicate (n = 3).

### 2.6. ABTS (2,2′-Azinobis-(3-ethylbenzthiazoline-6-sulphonate)) Assay

A stock solution of ABTS radical cation (ABTS⁺) was prepared by mixing ABTS (7 mmol/L) with potassium persulfate (2.45 mmol/L) following the method described by Wojdyło [75]. The mixture was allowed to stand at room temperature in the dark for 12–16 h prior to use. The stock solution was then diluted with ultrapure water to achieve an absorbance of 734 nm. For the assay, 0.01 mL of the sample was mixed with 0.19 mL of an ABTS⁺ solution. The mixture was incubated in the dark at room temperature for 10 min, after which the absorbance was measured at 734 nm using a spectrophotometer. Vitamin C was used as the standard. The percentage of ABTS reduction was calculated using the following formula:

% ABTS reduction = (1 − As/Ac) × 100

Ac = absorbance of control;

As = absorbance of the sample.

### 2.7. Ferric Reducing Antioxidant Power (FRAP) Assay

The analysis of total polyphenol concentrations was performed according to the modified method of Fernández-Arroyo [75]. First, an acetate buffer was prepared by dissolving 0.364 g of anhydrous sodium acetate and adding 3.2 mL of glacial acetic acid, then diluting with water to a final volume of 200 mL and adjusting the pH to 3.6. This buffer was then mixed with TPTZ (10 mmol/L) and FeCl_3_ (20 mmol/L) in a 10:1:1 ratio to prepare the FRAP stock solution. For the assay, 0.19 mL of FRAP reagent was mixed with 0.01 mL of the sample. The mixture was incubated in a water bath at 37 °C for 10 min, and the absorbance was measured at 593 nm using a spectrophotometer. The FRAP reagent sample solution was used as the blank.

### 2.8. Statistical Analysis

The experimental results are presented as the mean ± standard deviation. Data analysis was performed using SPSS 24.0 software and SigmaPlot 15.0 software. The normality of variance of all the data variables were tested using Shapiro–Wilk tests. One-way analysis of variance (ANOVA) with post hoc Duncan’s multiple range test was used to estimate the statistical significance among plant extracts combination and with dragon’s blood experiment treatments at *p* < 0.05. If the normality test failed, we used Kruskal–Wallis one way analysis of variance on rank testing at *p* < 0.05. All data presented in the text and figures are the mean ± standard deviation.

This study employed independent sample two-way analysis of variance and the significant level set as the p-value was lower than 0.05, with five substances, including water, 30% ethanol, 70% ethanol, 30% methanol, and 70% methanol as the A factor. Three different temperatures of 25 °C, 50 °C, and 75 °C were the B factor. The dependent variable was the antioxidant activity level (in percentage) of each sample extract for conducting the independent sample two-way analysis of variance. The analytical strategy involved first testing the significance of the interaction between factors A and B. If the interaction between the two factors was not significant, a comparison of the main effects was conducted by directly comparing the marginal means, yielding results similar to conducting independent sample one-way analysis of variance individually. If the interaction was significant, further comparative analysis of “simple main effects” was carried out, with the aim of comparing differences between fine-grained means. In the data analysis process, bootstrapping was employed with a sampling frequency set at 5000 times to estimate the study data and correct the data distribution to exhibit normality [76].

## 3. Results

### 3.1. The Optimal Antioxidant Activity Extraction Conditions of Natural Plants and Dragon’s Blood Extracts

We screened five different concentrations of ethanol and methanol at 30%, 50%, 70%, 95%, and 99.9% by the DPPH method. Here, we selected extracts obtained using 30%, 50%, and 70% ethanol and methanol solvents to conduct further experimentation. It was found that the activity of some of the extracts decreased as soon as they reached high temperatures, and the activity of some extracts using water was lower than that of other solutions.

We screened the optimal conditions of crude extraction from ifferent natural plants, *Hibiscus sabdariff* (roselle), *Clitoria ternatea* (butterfly pea flower), *Camellia sinensis* and *Pandanus amaryllifolius* (pandan). The flower of *Hibiscus sabdariffa* (roselle) was found to have good heat resistance and the extract was red. It was also found that heating roselles at different temperatures does not affect their activity when using either ethanol or methanol solvents. However, the activity of roselle in water extraction was significantly lower, and the highest activity was 93%. Hence, the conditions of 70% ethanol extraction at 50 °C were used as the conditions for the subsequent active extraction of *Hibiscus sabdariff* (roselle) (Supplementary Data). The extract of the flower of *Clitoria ternatea* (butterfly pea flower) has a blue color. Compared with roselle, the activity of butterfly pea flower is slightly lower. It can be found that the water extraction activity was the lowest, and the highest activity was 85%. The best effect resulted from extracting 30% ethanol at 25 °C as the follow-up butterfly pea flower. The conditions for the active extraction are shown in the Supplementary Data. The antioxidant activities of purchased commercial green tea extract and *Camellia sinensis* dry leaves were very high, both as high as 90% or more. This finding means that the different extraction conditions of the two extracts do not strongly affect the activity. The conditions for the subsequent active extraction of green tea were chosen to achieve the highest activity of 93%, with the extract and 70% ethanol at 25 °C as the conditions (Supplementary Data). The activity of *Pandanus amaryllifolius* (pandan) leaves was between 85–90% of the activity of the flowers and the butterfly pea flower. Pandan leaves obtained different extract colors in different extraction conditions. The higher the concentration of the solution, the greener the color of the plant. The conditions selected for subsequent experiments were 30% ethanol at 25 °C with an activity of 90.45% and 70% methanol at 25 °C, with an activity of 90.41% (Supplementary Data).

The best conditions for the crude extraction of *Hylocereus* sp. (red dragon fruit) flowers are given as follows. We found that the flowers of *Hylocereus* sp. (red dragon fruit) can be defined into five different types: *Hylocereus* sp. (red dragon fruit) flowers, namely, red dragon fruit flowers (dry), red dragon fruit petals (dry), red dragon fruit stamens (dry), red dragon fruit petals (wet), and red dragon fruit stamens (wet). We first examined which type had the best antioxidant activity. The results showed that the antioxidant activity of dried red dragon fruit flowers was much better than that of the undried red dragon fruit flowers. The 70% methanol and 70% ethanol conditions for red dragon fruit flowers (dry) were better than other conditions, with values of 78%.Finally, we selected 30% ethanol at 25 °C as the experimental condition for the red dragon fruit flowers (dry) (Supplementary Data). The antioxidant activity of the red dragon fruit petals (dry) was 79.9% using 70% methanol at 25 °C, and the second-best activity was using 30% ethanol at 25 °C, with a 79.24% activity. The two extraction conditions underwent follow-up experiments to see if they would affect the final activity (Supplementary Data). The antioxidant activity of the red dragon fruit stamens (dry) was better than that of the red dragon fruit petals (dry) and red dragon fruit flowers (dry). The extraction conditions selected in the next experiment were 70% ethanol at 50 °C, and the antioxidant activity was 87.37% (Supplementary Data), followed by the red dragon fruit petals (wet) and red dragon fruit stamens (wet), where their activity was very low. In the experiment, if there was mucus in the red dragon fruit flower without drying, it would affect the measurement result (Supplementary Data). Therefore, the red dragon fruit flowers (dried), red dragon fruit petals (dried), and red dragon fruit stamens (dry) were used in follow-up experiments.

The best conditions for the crude extraction of dragon’s blood are given as follows. We purchased a lot of commercialragon’s blood samples from traditional Chinese medicine stores and examined their antioxidant activities. Then, we chose the best two to perform the interaction examination with natural plant extracts. The antioxidation of the four commercial dragon’s blood samples, *Dracaena* 1, *Dracaena* 2, *Dracaena* 3, and *Dracaena* 4, did not exceed a 60% DPPH antioxidant activity whether in water, ethanol or methanol, where, as long as the temperature increases, the activity will decrease (Supplementary Data). The other three commercial dragon’s blood samples, *Dracaena* 5, *Dracaena* 6, and *Dracaena* 7, did not exceed 50% DPPH antioxidant activity (Supplementary Data). The above seven commercial dragon’s blood samples were kept at a temperature of 25 °C to result in the best extraction conditions. For *Dracaena* 8, with the concentration of the methanol and ethanol solution increased to 70% and the temperature increased to 75 °C, the activity was improved to 68%. For *Dracaena* 9, the 25 °C extraction condition was better than the other temperatures. The optimal extraction condition was 30% ethanol with a heating temperature of 25 °C, where the activity was up to 82%. In particular, for the Indonesian dragon’s blood, low concentrations of ethanol and low temperatures achieved high activity (Supplementary Data).

We identified three species of dragon’s blood (*Daemonorops draco*, *Dracaena cochinchinensis*, and *Dracaena cinnabari*) and tested their antioxidant activity. The extraction conditions of the Sword leaf dragon’s blood (*Dracaena cochinchinensis*) with a higher solution concentration resulted in better activity and the temperature had little effect. In comparison, the activity was lower when using water extraction and 30% methanolextraction. Therefore, we chose 70% ethanol heated at 25 °C and 70% methanol heated at 50 °C was used in follow-up experiments for comparison (Supplementary Data). For Yemen Socotra dragon’s blood (*Dracaena cinnabari*), although 70% ethanol was better than water and low concentrations of methanol and ethanol, the activity was less than 80%. The result found that 70% methanol extraction was the best, with an activity of 83% (Supplementary Data). The activity of the Indonesian dragon’s blood was above 80%. Methanol or ethanol extraction affected the activity due to the increase in temperature. For this species, 70% ethanol at 25 °C and 70% methanol heating at 25 °C were selected as the follow-up research conditions (Supplementary Data). Finally, after screening the samples of dragon’s blood, we selected the two species of dragon’s blood *Dracaena cochinchinensis* and *Daemonorops draco* for the interaction experiments, where extraction of both can be performed in the condition of 70% ethanol or 70% methanol.

### 3.2. Total Anthocyanin and Antioxidant Capacity of Compound Natural Plant Extracts

The data for the total antioxidant and anthocyanin content for each plant samples and dragon’s blood samples were obtained by the total anthocyanin concentration (TAC), total polyphenol concentration (TPC), 2,2′-azinobis-(3-ethylbenzthiazoline-6-sulphonate (ABTS), and ferric reducing antioxidant power (FRAP), as shown in the Supplementary Data.

#### 3.2.1. Total Anthocyanin Concentration (TAC) Assay

The total anthocyanin concentration (TAC) assay evaluates the concentration of polyphenols, where higher values indicate higher concentrations. The highest total polyphenol concentration for *Hibiscus sabdariffa* was 3743 mg GAE/100 g when extracted with 30% ethanol at 50 °C. When extracted with 70% ethanol at the same temperature, the concentration was slightly lower at 3181 mg GAE/100 g. *Clitoria ternatea* flower extraction with 30% ethanol at 25 °C yielded a concentration of 2951 mg GAE/100 g, which was comparable to that of *Hibiscus sabdariffa*.

In contrast, *Camellia sinensis* extracts exhibited consistently low polyphenol concentrations under various conditions, despite having a high overall polyphenol content. Notably, heating *Camellia sinensis* with 70% ethanol at 25 °C resulted in a significantly high concentration of 9840 mg GAE/100 g. *Pandanus amaryllifolius* showed concentrations of 1920 mg GAE/100 g with 30% ethanol at 25 °C and 1829 mg GAE/100 g with 70% methanol at 25 °C, with no significant difference between these conditions.

Red dragon fruit (*Hylocereus* sp.) petals and stamens (wet) had low total polyphenol content. For instance, the wet red dragon fruit flower contained 2311 mg GAE/100 g when extracted with water at 50 °C, whereas the dry petals had 1794 mg GAE/100 g under the same conditions. The dry petals exhibited lower polyphenol content compared to the dry flowers. Conversely, the dry stamens had higher concentrations, with water extraction at 50 °C yielding 3358 mg GAE/100 g.

In the case of dragon’s blood, polyphenol concentrations were generally low. However, *Dracaena cochinchinensis* showed the highest concentration of 4715 mg GAE/100 g when extracted with 70% methanol at 50 °C. Using 70% ethanol at 50 °C resulted in a concentration of 3712 mg GAE/100 g. The second-highest concentration for dragon’s blood was 2088 mg GAE/100 g with 30% ethanol at 50 °C in the seeds of *Daemonorops draco*. Overall, *Hylocereus* sp. had the highest polyphenol concentration, followed by *Dracaena cochinchinensis*.

These findings underscore the significant variability in polyphenol concentrations across different plant materials and extraction conditions, highlighting the importance of optimizing extraction methods for each specific application.

#### 3.2.2. Total Polyphenol Concentration (TPC) Determination

The concentration of total polyphenols tends to increase with higher sample values. For instance, the highest total polyphenol concentration observed for *Hibiscus sabdariffa* was 3743 mg GAE/100 g when heated with 30% ethanol at 50 °C, compared to 3181 mg GAE/100 g when extracted with 70% ethanol at the same temperature. Similarly, the concentration of polyphenols in *Clitoria ternatea* flower extracted with 30% ethanol at 25 °C was 2951 mg GAE/100 g. The difference in total polyphenols from the *Hibiscus sabdariffa* flower was significant. The total polyphenol concentration of the commercial green tea extract was low regardless of the extraction conditions. The overall concentration of green tea was very high. In the follow-up experiment conditions, 70% ethanol was heated at 25 °C to reach 9840 mg GAE/100 g, with an excellent total polyphenol content. The concentration of pandan leaves heated at 25 °C with 30% ethanol was 1920 mg GAE/100 g, while the concentration at 70% methanol at 25 °C was 1829 mg GAE/100 g. Notably, there was no significant difference in activity between the two extract concentrations. In addition, the total polyphenol contents of red dragon fruit petals (wet) and red dragon fruit stamens (wet) were very low. For example, the red dragon fruit flower (wet) contained 2311 mg GAE/100 g when extracted in water at 50 °C, while the red dragon fruit petals (dry) contained 1794 mg GAE/100 g when extracted in water at 50 °C, where the activity for the red dragon fruit petals (dry) were lower than those of the red dragon fruit flowers (dry). Meanwhile, the other two extract condition of the red dragon fruit stamens (dry) were higher, where water extraction at 50 °C resulted in 3358 mg GAE/100 g.

In dragon’s blood, the concentration of polyphenols was low, but in *Dracaena cochinchinensis*, the concentration of 70% methanol heated at 50 °C was 4715 mg GAE/100 g, the highest concentration among all samples. If used, the concentration of 70% ethanol heated at 50 °C was also 3712 mg GAE/100 g. The second highest concentration of dragon’s blood was the concentration of 2088 mg GAE/100 g in 30% ethanol heated at 50 °C in the seeds of *Dracaena cochinchinensis*. In terms of overall data, *Camellia sinensis* had the highest concentration, followed by *Dracaena cochinchinensis*.

#### 3.2.3. 2,2′-Azinobis-(3-ethylbenzthiazoline-6-sulphonate (ABTS) Determination

From the data, it is evident that the antioxidant capacities of natural plants such as *Hibiscus sabdariffa*, the *Clitoria ternatea* flower, commercial green tea extract products, *Camellia sinensis*, and *Pandanus amaryllifolius* leaves are very high across various extraction conditions, with all exceeding 95% antioxidant activity, indicating excellent efficacy.

In contrast, the antioxidant activity of dried red dragon fruit flowers was 87% when extracted with 30% ethanol at 25 °C. Increasing the extraction temperature to 50 °C and 75 °C improved the activity to 90% and 92%, respectively, suggesting that higher temperatures enhance the ABTS activity of dried red dragon fruit flowers. For dried red dragon fruit petals, both water extraction at 75 °C and 30% ethanol extraction at 50 °C yielded an activity of 86%. Dried red dragon fruit stamens displayed robust antioxidant activity under various extraction conditions, achieving 94% activity with 70% ethanol at 50 °C. Furthermore, a comparison between the antioxidant activities of wet red dragon fruit petals and wet red dragon fruit stamens revealed that dried red dragon fruit stamens exhibited the highest activity among the tested samples. Conversely, wet red dragon fruit petals had the lowest activity, falling below 70%. These results underscore the influence of extraction conditions on the antioxidant capacities of different plant materials, highlighting the importance of optimizing these parameters for maximum efficacy.

Among the dragon’s blood samples, the Jingji brand, Jinxing brand, Baozhu brand, Crown brand, Xianfeng brand, Hong Kong orchid brand, Chungyaon Qilin brand, Baozhu brand, and Qilin brand exhibited lower activity levels. The Yemen Socotra dragon’s blood, when heated at 75 °C with 70% ethanol, demonstrated the highest activity at 94%. Similarly, the Indonesian dragon’s blood, when treated with 70% ethanol at 25 °C, exhibited an activity level of 89%. However, the activity with water extraction was notably lower, with an activity of only 17% at 25 °C. Among all samples, *Dracaena cochinchinensis* showed the highest ABTS activity, reaching 97% when heated with 70% ethanol at 50 °C, and 95% when heated with 70% methanol at the same temperature. The activity of *Daemonorops draco* seeds was 87% when treated with 70% ethanol at 25 °C, while it dropped to 44% when treated with 70% methanol at the same temperature.

#### 3.2.4. Ferric Reducing Antioxidant Power (FRAP) Determination

The ferric reducing antioxidant power experiment measures the reduction concentration, where higher values indicate higher concentrations. In subsequent experiments, the extraction condition for *Hibiscus sabdariffa* (roselle) involved 70% ethanol heated at 50 °C, resulting in a concentration of 3.05 mg/mL, demonstrating a high reduction ability. *Clitoria ternatea* (butterfly pea flower) extracted with 30% ethanol at 50 °C yielded a concentration of 2.09 mg/mL, which was optimal for this plant, although slightly lower than the reduction concentration for *Hibiscus sabdariffa*.

The highest total content among all natural plants was observed in *Camellia sinensis*, with a concentration of 3.85 mg/mL. In contrast, the commercial green tea extract had approximately half the reduction concentration of natural green tea. *Pandanus amaryllifolius* (pandan leaves) achieved a maximum concentration of 1.03 mg/mL when extracted with 30% ethanol at 50 °C. Among the red dragon fruit flowers, the highest concentration for the dried flower was 1.07 mg/mL with 70% ethanol heated to 75 °C. The highest concentration for dried red dragon fruit petals was slightly lower, at 0.96 mg/mL.

Dried red dragon fruit flowers exhibited the highest activity, whereas both wet and dry red dragon fruit stamens reached a maximum concentration of 0.96 mg/mL, indicating the lowest activity among the samples.

For Socotra dragon’s blood, the highest content was 2.17 mg/mL when extracted with 70% ethanol at 25 °C. Indonesian dragon’s blood, extracted with 70% methanol at 50 °C, had a concentration of 1.42 mg/mL. *Daemonorops draco* seeds, extracted with 30% ethanol at 50 °C, achieved a content of 1.30 mg/mL. Conversely, *Dracaena cochinchinensis* had a concentration of 0.90 mg/mL when extracted with 70% methanol at 50 °C, with the remaining dragon’s blood samples having very low concentrations, which were almost negligible.

These findings highlight the variability in the reduction concentrations across different plant materials and extraction conditions, emphasizing the need to optimize the extraction parameters for each specific application.

### 3.3. Research on Compound Extracts of Natural Plants and Dragon’s Blood

Plant ethanol extraction was performed with 70% ethanol extraction at 50 °C. The experiment was classified into three categories: natural plant compound extracts, natural plant compound extracts + *Daemonorops draco*, and natural plant compound extracts + *Dracaena cochinchinensis*. In the follow-up experiments, we compared the differences and selected several composite extract samples to study the maintenance of composite extract activity.

#### 3.3.1. Screening Results of Natural Plant Compound Extracts

**Figure 1:**
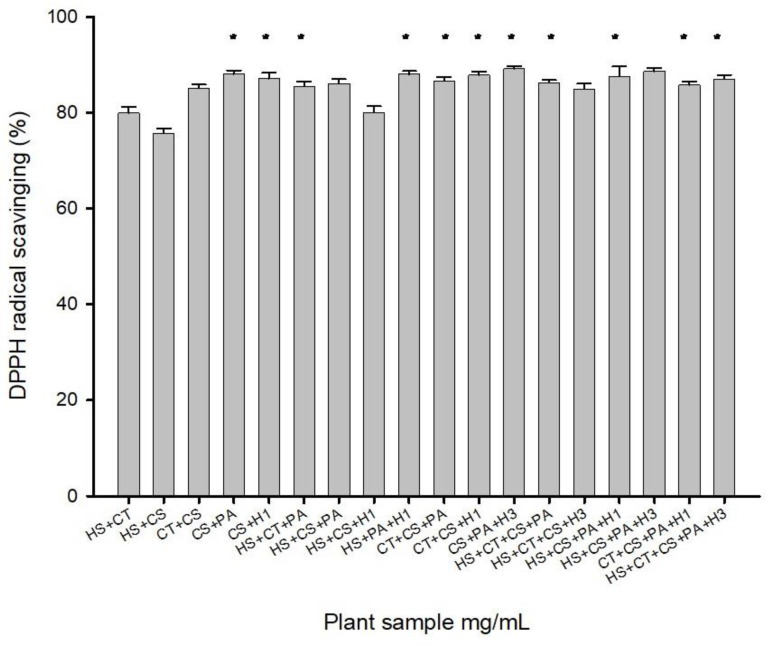
Screening results of complex extracts from natural plants using ethanol conditions (n = 30). Columns marked with an asterisk (*) indicate samples that are significantly different (*p* < 0.05). Abbreviations: HS, *Hibiscus sabdariffa*, Roselle; CT, *Clitoria ternatea*, butterfly pea flower; GE, commercial green tea extract; CS, *Camellia sinensis*, green tea; PA, *Pandanus amaryllifolius*, pandan leaves; H1, dry *Hylocereus* sp., red dragon’s fruit flowers (dry); H2, dry *Hylocereus* sp. petals, red dragon’s fruit flower petals (dry); H3, dry *Hylocereus* sp. stamens, red dragon’s fruit flower stamens (dry); H4, wet *Hylocereus* sp. petals, red dragon’s fruit flower petals (wet); H5, wet *Hylocereus* sp. stamens, red dragon’s fruit flower stamens (wet).

The natural plant extracts were initially extracted using ethanol. The combination of *Camellia sinensis* and *Pandanus amaryllifolius* leaves exhibited the highest activity at 89.45%, whereas the combination of *Hibiscus sabdariffa* and *Camellia sinensis* had the lowest activity at 76.38% (Figure 1). These results indicate that the relative activities of the extracts remained consistent, regardless of whether ethanol or the optimal extraction conditions were used.

Furthermore, it was observed that the *Hibiscus sabdariffa* and *Camellia sinensis* extracts showed higher activity when the optimal extraction conditions were employed compared to ethanol extraction alone. Based on the experimental results, the screening of natural plant compound extracts identified the following combinations for their high activities: *Camellia sinensis* + *Pandanus amaryllifolius* leaves, *Camellia sinensis* + dried red dragon fruit flowers, *Hibiscus sabdariffa* + *Clitoria ternatea* + *Pandanus amaryllifolius* leaves, butterfly pea + green tea + *Pandanus amaryllifolius* leaves, and *Camellia sinensis* + *Pandanus amaryllifolius* leaves. Additionally, the combinations of dried red dragon fruit stamens, *Hibiscus sabdariffa* + *Camellia sinensis* + *Pandanus amaryllifolius* leaves + dried red dragon fruit stamens, *Clitoria ternatea* flowers + *Camellia sinensis* + *Pandanus amaryllifolius* leaves + dried red dragon fruit stamens, and *Hibiscus sabdariffa* + *Clitoria ternatea* flowers + *Camellia sinensis* + *Pandanus amaryllifolius* leaves + dried red dragon fruit stamens were selected as the eight pairing methods.

#### 3.3.2. Screening Results of the Compound Extracts of Natural Plants and the Seeds of *Daemonorops draco*

**Figure 2:**
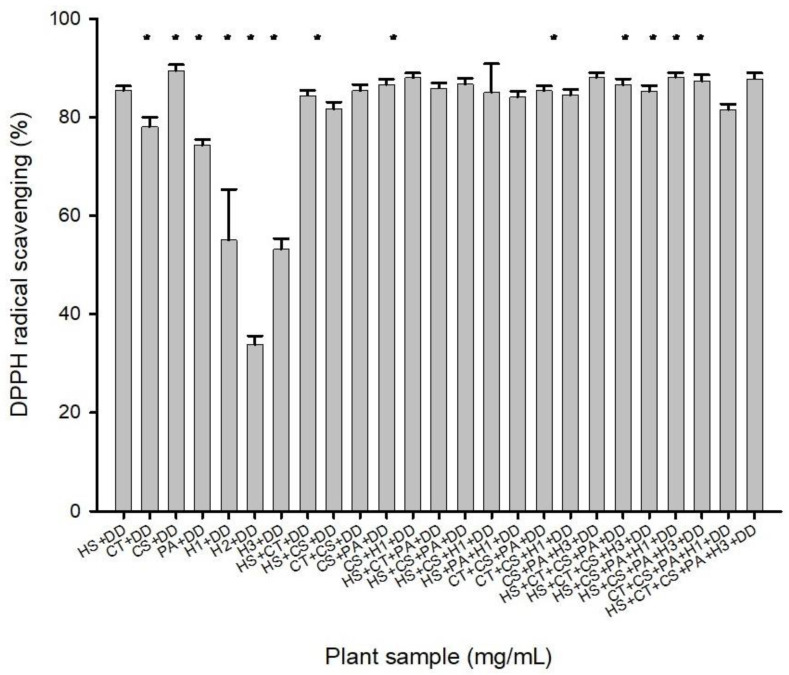
Screening results of complex extracts from natural plants with *Daemonorops draco* using ethanol conditions (n = 30). Columns marked with an asterisk (*) indicate samples that are significantly different (*p* < 0.05). Abbreviations: HS, *Hibiscus sabdariffa*, Roselle; CT, *Clitoria ternatea*, butterfly pea flower; GE, commercial green tea extract; CS, *Camellia sinensis*, green tea; PA, *Pandanus amaryllifolius*, pandan leaves; H1, dry *Hylocereus* sp., red dragon’s fruit flowers (dry); H2, dry *Hylocereus* sp. petals, red dragon’s fruit flower petal (dry); H3, dry *Hylocereus* sp. stamens, red dragon’s fruit flower stamens (dry); H4, wet *Hylocereus* sp. petals, red dragon’s fruit flower petals (wet); H5, wet *Hylocereus* sp. stamens, red dragon’s fruit flower stamens (wet); DD, *Daemonorops draco*.

Natural plant extracts were compounded with *Daemonorops draco* and extracted with ethanol. The combination of *Camellia sinensis* and *Daemonorops draco* exhibited the highest activity at 91.36%. The combination of *Hibiscus sabdariffa*, *Clitoria ternatea*, *Camellia sinensis*, *Pandanus amaryllifolius* leaves, and wet red dragon fruit stamens had an activity of 89%, making it the second highest (Figure 2). The data indicate that the combination of dried red dragon fruit petals and *Daemonorops draco* had the lowest composite activity, with 35.62% in ethanol. Overall, the optimal combination was *Camellia sinensis* and *Daemonorops draco*. Based on the experimental results, the following combinations were selected for their high composite activity in the screening of natural plant extracts: 1. *Camellia sinensis* + *Daemonorops draco*; 2. *Camellia sinensis* + *Pandanus amaryllifolius* leaves + *Daemonorops draco*; 3. *Camellia sinensis* + dried red dragon fruit flowers + *Daemonorops draco*; 4. *Hibiscus sabdariffa* + *Clitoria ternatea* + *Pandanus amaryllifolius* leaves + *Daemonorops draco*; 5. *Clitoria ternatea* + *Camellia sinensis* + *Pandanus amaryllifolius* leaves + *Daemonorops draco*; 6. *Camellia sinensis* + *Pandanus amaryllifolius* leaves + dried red dragon fruit stamens + *Daemonorops draco*; 7. *Hibiscus sabdariffa* + *Camellia sinensis* + *Pandanus amaryllifolius* leaves + dried red dragon fruit stamens + *Daemonorops draco*; 8. *Clitoria ternatea* + *Camellia sinensis* + *Pandanus amaryllifolius* leaves + dried red dragon fruit flowers + *Daemonorops draco*; 9. *Hibiscus sabdariffa* + *Clitoria ternatea* + *Camellia sinensis* + *Pandanus amaryllifolius* leaves + dried red dragon fruit stamens + *Daemonorops draco*. These nine combinations demonstrated the highest potential for antioxidant activity.

#### 3.3.3. Screening Results of Compound Extracts of Natural Plants and *Dracaena cochinchinensis*

**Figure 3:**
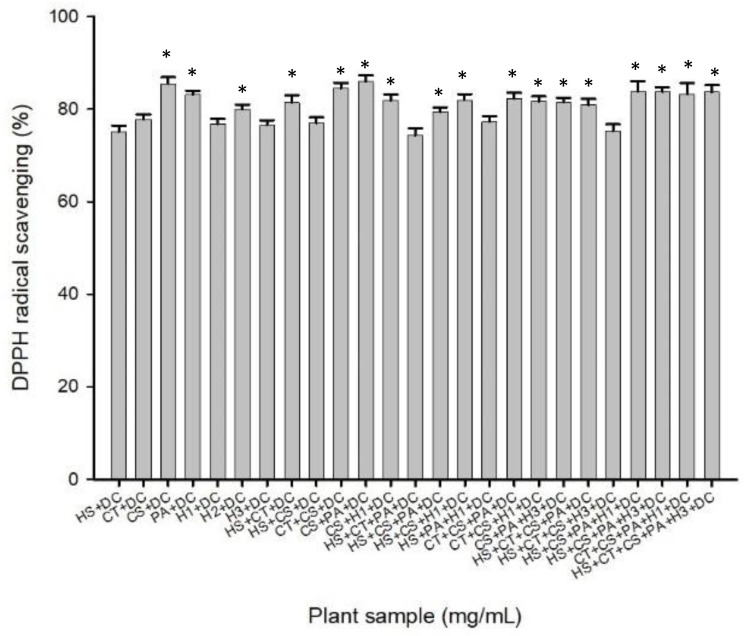
Screening results of complex extracts from natural plants with *Dracaena cochinchinensis* using ethanol conditions (n = 30). Columns marked with an asterisk (*) indicate samples that are significantly different (*p* < 0.05). Abbreviations: HS, *Hibiscus sabdariffa*, Roselle; CT, *Clitoria ternatea*, butterfly pea flower; GE, commercial green tea extract; CS, *Camellia sinensis*, green tea; PA, *Pandanus amaryllifolius*, pandan leaves; H1, dry *Hylocereus* sp., red dragon’s fruit flowers (dry); H2, dry *Hylocereus* sp. petals, red dragon’s fruit flower petals (dry); H3, dry *Hylocereus* sp. stamens, red dragon’s fruit flower stamens (dry); H4, wet *Hylocereus* sp. petals, red dragon’s fruit flower petals (wet); H5, wet *Hylocereus* sp. stamens, red dragon’s fruit flower stamens (wet); DC, *Dracaena cochinchinensis*.

Natural plant extracts were compounded with *Dracaena cochinchinensis* and extracted with ethanol. The combination of *Camellia sinensis* and *Dracaena cochinchinensis* exhibited the highest activity at 84.39%, while the combination of *Hibiscus sabdariffa*, *Clitoria ternatea* flowers, *Clitoria ternatea* leaves, and *Dracaena cochinchinensis* had the lowest activity (Figure 3). Based on the experimental results, the following nine combinations were selected for their potential: 1. *Camellia sinensis* + *Dracaena cochinchinensis*; 2. *Camellia sinensis* + *Clitoria ternatea* leaves + *Dracaena cochinchinensis*; 3. *Camellia sinensis* + dried red dragon fruit flowers + *Dracaena cochinchinensis*; 4. *Hibiscus sabdariffa* + *Clitoria ternatea* + *Pandanus amaryllifolius* leaves + *Dracaena cochinchinensis*; 5. *Clitoria ternatea* + *Camellia sinensis* + *Pandanus amaryllifolius* leaves + *Dracaena cochinchinensis;* 6. *Camellia sinensis* + *Pandanus amaryllifolius* leaves + dried red dragon fruit stamens + *Dracaena cochinchinensis*; 7. *Hibiscus sabdariffa* + *Camellia sinensis* + *Pandanus amaryllifolius* leaves + dried red dragon fruit stamens + *Dracaena cochinchinensis*; 8. *Clitoria ternatea* flowers + *Camellia sinensis* + *Pandanus amaryllifolius* leaves + dried red dragon fruit flowers + *Dracaena cochinchinensis*; 9. *Hibiscus sabdariffa* + *Clitoria ternatea* flowers + *Camellia sinensis* + *Pandanus amaryllifolius* leaves + dried red dragon fruit stamens + *Dracaena cochinchinensis.* These nine combinations were identified for further investigation based on their activity levels.

### 3.4. Total Anthocyanin and Antioxidant Capacity of Compound Natural Plant Extracts with Dragon’s Blood

Natural extracts increased or decreased the concentrations and activities of total anthocyanins and antioxidant capacity after compounding (Table 1).

#### 3.4.1. Determination Results of Total Anthocyanin Concentration (TAC)

Based on the experimental findings, in the ethanol extraction phase, each compound exhibited a distinct anthocyanin content. Among these, the combination of *Hibiscus sabdariffa*, *Clitoria ternatea*, and *Pandanus amaryllifolius* leaves had the highest anthocyanin content at 1926 mg CGE/100 g. Following closely, the combination of *Hibiscus sabdariffa*, *Clitoria ternatea*, *Pandanus amaryllifolius* leaves, and dry red dragon fruit stamens was recorded at 1199 mg CGE/100 g. Meanwhile, the combination of *Hibiscus sabdariffa*, *Clitoria ternatea*, *Pandanus amaryllifolius* leaves, and *Dracaena cochinchinensis* yielded 1075 mg CGE/100 g. Conversely, the combination of *Hibiscus sabdariffa* flowers, *Clitoria ternatea* flowers, and *Pandanus amaryllifolius* leaves had the lowest content, while *Camellia sinensis*, dry red dragon fruit flowers, and *Daemonorops draco* showed only 3 mg CGE/100 g. Notably, the highest anthocyanin content was observed in the *Hibiscus sabdariffa*, *Clitoria ternatea*, and *Pandanus amaryllifolius* leaves combination, reaching 1765 mg CGE/100 g. Following this, the combination of *Hibiscus sabdariffa*, *Clitoria ternatea*, *Pandanus amaryllifolius* leaves, and dry red dragon fruit stamens displayed the second-highest content at 1256 mg CGE/100 g. Interestingly, two compound extracts exhibited no cyanine content: *Camellia sinensis*, dry red dragon fruit flowers, and *Daemonorops draco*, along with *Clitoria ternatea* flowers and *Dracaena cochinchinensis*. Additionally, when combined with the *Hibiscus sabdariffa* extract, there was a notable increase in the total anthocyanin concentration.

#### 3.4.2. Determination Results of Total Polyphenol Concentration (TPC)

The total polyphenol concentration (TPC) is an indicator of the content of polyphenolic compounds in plant extracts. Polyphenolic compounds are important bioactive components in plants, possessing potent biological activities, such as antioxidant, anti-inflammatory, and anti-inflammatory effects. Therefore, measuring TPC is crucial for assessing the nutritional value and health benefits of plant extracts. In ethanol extraction, the combination of *Camellia sinensis* and *Dracaena cochinchinensis* exhibited the highest concentration at 10,500 mg GAE/100 g, followed closely by *Camellia sinensis* combined with dry red dragon fruit flowers, which recorded a concentration of 10,090 mg GAE/100 g. Conversely, the lowest concentration was observed in the combination of *Hibiscus sabdariffa*, *Clitoria ternatea* flowers, *Pandanus amaryllifolius* leaves, and *Daemonorops draco*, registering at 2967 mg GAE/100 g.

#### 3.4.3. Determination Results of ATBS

The analysis of the free radical scavenging ability in ethanol extraction found that the activity of *Camellia sinensis* + *Daemonorops draco* was 93%. In addition, four combinations had the lowest activity: *Camellia sinensis* + *Pandanus amaryllifolius* leaves + red dragon fruit stamens (dry), *Hibiscus sabdariffa* + *Camellia sinensis* + *Pandanus amaryllifolius* leaves + red dragon fruit stamens (dry) + *Dracaena cochinchinensis*, *Clitoria ternatea* flowers + *Camellia sinensis* + *Pandanus amaryllifolius* leaves + red dragon fruit stamens (dry) + *Dracaena cochinchinensis*, and *Hibiscus sabdariffa* + *Clitoria ternatea* + *Camellia sinensis* + *Pandanus amaryllifolius* leaves + red dragon fruit stamens (dry) + *Dracaena cochinchinensis*, which all had an activity of 86%. The above data show that adding more extracts to the composite extract would not completely increase the activity.

#### 3.4.4. Determination Results of FRAP

Among the ethanol extraction conditions, the total antioxidant content of the combination of *Hibiscus sabdariffa*, *Clitoria ternatea*, *Pandanus amaryllifolius* leaves, and *Daemonorops draco* was 1.8 mg/mL, which was the lowest recorded. This was followed by the combination of *Hibiscus sabdariffa*, *Clitoria ternatea*, *Pandanus amaryllifolius* leaves, and *Dracaena cochinchinensis*, which measured 2 mg/mL. Notably, when dragon’s blood was not included, the combination of *Hibiscus sabdariffa*, *Clitoria ternatea*, and *Pandanus amaryllifolius* leaves displayed a relatively high content of 2.3 mg/mL.

Under the best extraction conditions, the combinations with lower activity were those involving *Daemonorops draco* and *Dracaena cochinchinensis*, with contents of 1.86 mg/mL and 1.83 mg/mL, respectively. Interestingly, the use of ethanol or optimal extraction conditions did not significantly impact the total antioxidant content. However, the composition of the compound extract played a crucial role.

After those tests, it was observed that different extraction conditions did not substantially affect the contents and concentrations of the extracts; however, the combination of different plants significantly influenced the final results, particularly in terms of anthocyanin content and antioxidant activity. It was noted that even when anthocyanin content was low, certain extracts still exhibited high antioxidant activity. Similarly, extracts with minimal total polyphenol content could still display high activity.

This experiment underscores the importance of considering the varied compositions of compound extracts in determining their content and concentration.

## 4. Discussion

### 4.1. Dragon’s Blood and Plant Extracts Using Different Extraction Conditions in DPPH and Four Other Assays

Previous studies on natural substances have predominantly focused on single plants to explore their activity and application value; however, the activity of a single plant may not meet the needs of agriculture industry and cosmetics developers. Therefore, this study aims to use compound plant materials, integrating five natural plants, to evaluate their various activities and investigate their potential applications for the traditional Chinese medicine dragon’s blood. The obtained extracts will be further evaluated for their antioxidant capacity. Following screening, the compound extracts will be studied for their activity to explore whether the composite plant materials exhibit synergistic effects, aiming to assess their feasibility for application in the traditional Chinese medicine dragon’s blood.

#### 4.1.1. Dragon’s Blood Extraction and Selections

The results of determining dragon’s blood activity using DPPH and ABTS assays were consistent. For instance, water extraction at 25 °C yielded a higher concentration, but the total activity was only 60%. *Daemonorops draco* was best extracted with 70% ethanol and 70% methanol to achieve higher activity. The TAC study showed little to no total anthocyanin content, with only some assays detecting very small amounts. *Dracaena cochinchinensis* exhibited the best extraction results with 70% ethanol and 70% methanol, while *Daemonorops draco* showed the highest content with a 30% ethanol extract. Overall, the dragon’s blood extracts demonstrated potent antioxidant properties as measured by DPPH, ABTS, FRAP, and TPC assays.

Previous studies have demonstrated that dragon’s blood exhibits a good antioxidant activity when extracted with methanol [16]. Additionally, extraction studies related to dragon’s blood have utilized both methanol and ethanol as extraction solvents. Therefore, this study extracted dragon’s blood under various conditions to compare the results.

There was a noticeable difference in the dissolution behavior between samples from different sources during the pre-extraction handling. For example, dragon’s blood powders from the Jinji, Jingxin, Crown, and Bouzhu brands did not completely dissolve in the extraction solution. Particularly, Crown brand powders floated on the surface when extracted with water. When using 30% ethanol, the powders became viscous and did not fully dissolve. In contrast, powders from Xianfeng, Hong Kong’s Orchid, and Chungyaon Qilin dragon’s blood did not adhere to the bottom or walls of the centrifuge tube, but their colors were very light after different extraction conditions. The Baozhu brand, using 30% ethanol and 70% methanol, adhered to the tube walls and exhibited a colorless to light yellow hue.

Yemen Socotra dragon’s blood did not stick to the centrifuge tube. Indonesian dragon’s blood, when extracted with 70% ethanol or 70% methanol, produced a dark red color and did not become sticky during extraction. Specifically, the 70% ethanol extraction solution was dark red, while other conditions resulted in a dark yellow color. During the extraction of *Daemonorops draco*, the samples did not stick to the centrifuge tube, however, with 30% ethanol and 70% methanol, slight adhesion to the tube’s bottom and walls was observed. *Dracaena cochinchinensis* extracted with 70% ethanol and 70% methanol yielded a deep red color.

The activity of dragon’s blood was correlated with the color of the extract, where darker colors indicated higher activity. The initial extraction conditions resulted in the highest activity for *Daemonorops draco*. It was found that when natural plants were compounded with *Daemonorops draco*, the combination with certain extracts produced the highest activity. Notably, *Daemonorops draco*, which had a lighter seed coat, exhibited high antioxidant activity due to the seed coat’s properties.

#### 4.1.2. Natural Plant Extraction

In DPPH, previous research [77] has shown that the red pigment of *Hibiscus sabdariffa* is the main source of its antioxidant capacity. Predecessors have used 30% ethanol to conduct experiments and obtained good antioxidant activity. In our study, *Hibiscus sabdariffa* was extracted with 30% and 70% ethanol and 30% and 70% methanol for experiments, with the heating temperatures of 25 °C, 50 °C, and 75 °C. The extracts showed stable antioxidant activity. The results had excellent antioxidant activity, consistent with the previous research. Only the water extraction of roselle had low antioxidant activity. *Hibiscus sabdariffa* had good antioxidant activity by TAC, TPC, ABTS, and FRAP. Among all data, the extraction results under different conditions were consistent and had the best activity and concentration. The results of the total anthocyanin concentration of roselle extract in this study and those of related research were consistent. Therefore, we conclude that *Hibiscus sabdariffa* anthocyanins are a good source. Previous researchers have pointed out that *Hibiscus sabdariffa* and its extracts have functional properties that can be used to develop new products with additional nutritional properties that provide health benefits [8,25].

*Clitoria ternatea* flowers in DPPH, using 30% and 70% ethanol and methanol, showed better activity than that with water extraction. In the previous research of Escher et al. [78], *Clitoria ternatea* flowers showed 55–56% activity when extracted with water, which was lower than our experiments (74–76% activity). TAC content was extracted with water at 60 °C for 45 min to obtain a content of 395 mg CGE/100 g. Meanwhile, in this experiment, the content of 205 mg CGE/100 g was obtained by extracting with water at 50 °C for 20 min. The differences may come from different extraction times and temperatures. In the FRAP analysis, the previous study stated that the highest content could be obtained by extracting at 60 °C for 15 min. In our experiment, the temperatures were 25 °C, 50 °C, and 75 °C, and the extraction time was 20 min. According to the data of this study, the highest content of FRAP can be obtained at 50 °C. In this study, TPC extracted with water had a better polyphenol content, while the extraction with 70% ethanol had a lower content. ABTS showed a consistent radical scavenging ability, slightly lower than 30% methanol extraction at 25 °C. Studies in the literature reported that the differences between content values might be related to the different extraction and quantification methods for *Clitoria ternatea* flowers [79].

Masek [80] showed that *Camellia sinensis* extract had a 93.6% scavenging activity for free radicals in ABTS and 78.3% for DPPH. The average activity of chemical *Camellia sinensis* extract in ABTS in this study was 98.34%, while the average activity of DPPH was 91.28%. The average activity of natural *Camellia sinensis* extract in ABTS was 98.17%, while the average activity of DPPH was 92.8%. Compared with the previous literature, the activity shown in our study was higher; however, it must be noted that there are many varieties of *Camellia sinensis*, and thus, many factors may lead to different activities. In our study, there were consistent differences in the TAC, TPC, and FRAP between the commercial green tea extract and the *Camellia sinensis* extract. Overall, the content of the *Camellia sinensis* extract was much higher than that of the commercial green tea extract, and the commercial one contained a slight number of total anthocyanins. Spectrophotometric studies in the previous literature showed that the anti-free radical activity increased with the increase of the compound concentration, and *Camellia sinensis* extract was confirmed to have strong antioxidant activity [80].

Jiamthaisong and Krisdaphong [81] used a DPPH method to measure the free radical scavenging activity of the leaves and roots of *Pandanus amaryllifolius* leaf extracts with 95% methanol and polyethylene glycol (PEG). Meanwhile, previous studies, such as that of Suwannakul [82], showed higher activity in the two solvents ofethanol andwater; however acetone, and ethyl acetate, showed the lowest activity. Therefore methanol, ethanol, and water were used to extract *Pandanus amaryllifolius* leaves in our study, whose antioxidant activities were 86.29–90.45%. In addition, Suwannakul [83] proposed using the leaves of *Pandanus amaryllifolius* and showed that the content of TPC in ethanol was higher than that in water or methanol extracts [83]. In our study, the content of TPC was not much different. At present, there is no relevant academic research on the ABTS, FRAP, and TAC of pandan leaves. Our study showed no total anthocyanin content, and the performance of ABTS and FRAP was consistent with the results of DPPH and TPC. Previous studies have shown that many variables, such as the extraction time, extraction method, temperature, and solvent, account for the differences [83].

Jerônimo [84] studied the peel and pulp of pitaya, indicating that when DPPH was measured, the antioxidant activity of the pitaya peel through methanol extraction was higher than that in the pulp. According to other studies, the total phenolic and flavonoid content in the pitaya fruit pulp and peel were similar, indicating that the pulp and peel are rich in polyphenols and are good sources of antioxidants [55]. In our study, the red dragon fruit flower was used for experimentation. Overall, the activity of dried red dragon fruit flowers was better than that of the wet red dragon fruit flowers. According to the results of DPPH, ABTS, and FRAP, ethanol and methanol extraction had good activity, among which stamen activity was the best. After drying in TAC, the red dragon fruit flowers, red dragon fruit petals, and red dragon fruit stamens were extracted with 70% ethanol and methanol, all with anthocyanin content, and the TPC content was the best for water extraction.

### 4.2. Compound Extracts in DPPH and Other Four Assays

For the complex extract of *Daemonorops draco*, we observed that combining red dragon fruit flowers (dry) with *Daemonorops draco* exhibited the highest antioxidant activity, with an optimal extract showing 76.28% activity compared to only 58.76% with ethanol extraction. This significant difference highlights the variability in antioxidant activity based on extraction methods. Other compound extracts displayed similar trends. The combination of red dragon fruit petals (dry) and red dragon fruit stamens (dry) with *Daemonorops draco* had the lowest activity, suggesting that these specific extracts are not suitable for combination with *Daemonorops draco* individually. However, when other extracts were compounded with *Daemonorops draco*, they maintained similarly high activity levels. Notably, the combination with *Clitoria ternatea* flower showed lower ethanol extraction activity than the optimal extraction, unlike other natural plant extracts.

Regarding total anthocyanin content (TAC), dragon’s blood had low or no anthocyanin content, resulting in lower measured contents in compounds including *Daemonorops draco*. However, *Hibiscus sabdariffa* + *Clitoria ternatea* flower + *Pandanus amaryllifolius* leaves + *Daemonorops draco* exhibited the highest content. Even without *Daemonorops draco*, this combination showed high anthocyanin levels. In terms of total polyphenol content (TPC), *Camellia sinensis* + *Daemonorops draco* had the highest concentration, whereas *Hibiscus sabdariffa* + *Clitoria ternatea* flowers + *Pandanus amaryllifolius* leaves + *Daemonorops draco* had the lowest. For ABTS, the activity increased slightly with the addition of dragon’s blood, and the ethanol extraction results were consistent, with optimal extraction activity. The most significant difference in FRAP was observed with *Hibiscus sabdariffa* + *Clitoria ternatea flower* + *Pandanus amaryllifolius* leaves + *Daemonorops draco*, which had the lowest activity compared to other extracts, highlighting a unique finding.

Regarding the complex extracts with *Dracaena cochinchinensis*, we compared the DPPH and ABTS results. There was little difference between ethanol extraction and the optimal extraction, but the overall activity was higher than that of the compound extracts with *Daemonorops draco*. The TAC content was also higher than the compound extracts with *Daemonorops draco*, although slightly lower than the compounds without added dragon’s blood. For TPC, the compound of *Hibiscus sabdariffa* + *Clitoria ternatea* flowers + *Pandanus amaryllifolius* leaves + *Dracaena cochinchinensis* had the lowest content. In FRAP determination, the content was comparable to that of the compound extracts without *Dracaena cochinchinensis* or *Daemonorops draco*, indicating a robust iron reduction capacity, regardless of presence of dragon’s blood.

There were no significant statistical differences in the presence or absence of compound dragon’s blood ingredients, whether extracted with methanol or ethanol. After conducting these assays, we confirmed the active contents of different compound extracts. We discovered that increasing the number of plants in the compound does not necessarily enhance activity and that it may maintain or reduce it. For instance, the activity of the extracts with or without dragon’s blood was similar, but compounding with *Dracaena cochinchinensis* slightly reduced the activity.

For the complex extract of *Daemonorops draco*, we observed that the red dragon fruit flower (dry) in combination with *Daemonorops draco* exhibited the highest antioxidant activity, with an optimal extract showing 76.28% activity, compared to only 58.76% with ethanol extraction. This significant difference indicates the variability in antioxidant activity based on extraction methods. Other compound extracts displayed similar trends. The combination of red dragon fruit petals (dry) and red dragon fruit stamens (dry) with *Daemonorops draco* had the lowest activity, suggesting that these specific extracts are not suitable for combination with *Daemonorops draco* individually. However, when five other kinds of extract were compounded with *Daemonorops draco*, they maintained similar high activity levels. Notably, combination with *Clitoria ternatea* flowers showed lower ethanol extraction activity than the optimal extraction, unlike other natural plant extracts.

For total anthocyanin content (TAC), dragon’s blood had low or no anthocyanin content, resulting in lower measured contents in compounds including *Daemonorops draco*. However, *Hibiscus sabdariffa* + *Clitoria ternatea* flowers + *Pandanus amaryllifolius* leaves + *Daemonorops draco* exhibited the highest content. Even without *Daemonorops draco*, this combination showed high anthocyanin levels. In terms of the total polyphenol content (TPC), *Camellia sinensis* + *Daemonorops draco* had the highest concentration, whereas *Hibiscus sabdariffa* + *Clitoria ternatea* flowers + *Pandanus amaryllifolius* leaves + *Daemonorops draco* had the lowest. For ABTS, the activity increased slightly with the addition of dragon’s blood, and the ethanol extraction results were consistent with the optimal extraction activity. The most significant difference in FRAP was observed with *Hibiscus sabdariffa* + *Clitoria ternatea* flowers + *Pandanus amaryllifolius* leaves + *Daemonorops draco,* which had the lowest activity compared to other extracts, highlighting a unique finding.

Regarding the complex extracts with *Dracaena cochinchinensis*, we compared the DPPH and ABTS results. There was little difference between ethanol extraction and optimal extraction, but the overall activity was higher than that of the compound extracts with *Daemonorops draco*. The TAC content was also higher than the compound extracts with *Daemonorops draco*, although slightly lower than of the compounds without added dragon’s blood. For TPC, the compound of *Hibiscus sabdariffa* + *Clitoria ternatea* flowers + *Pandanus amaryllifolius* leaves + *Dracaena cochinchinensis* had the lowest content. In FRAP determination, the content was comparable to that of the compound extracts without *Dracaena cochinchinensis* or *Daemonorops draco*, indicating a robust iron reduction capacity regardless of the presence of dragon’s blood.

There was no significant statistical difference in the presence or absence of compound dragon’s blood ingredients, whether extracted with methanol or ethanol. After conducting these assays, we confirmed the active contents of different compound extracts. We discovered that increasing the number of plants in the compound does not necessarily enhance activity and that it may maintain or reduce it. For instance, the activities of the extracts with or without dragon’s blood were similar, but compounding with *Dracaena cochinchinensis* slightly reduced activity.

## 5. Conclusions

This study underscores the potent antioxidant activity achieved through the combination of diverse extracts. The results also demonstrate the antioxidant activities of these plant extract combinations for the first time. Regardless of whether the extract is utilized under optimal conditions or standard ethanol extraction, its impact on determining the composite extract remains minimal. Conversely, singular extracts exhibit superior performance in activity preservation. Particularly, the combination of *Camellia sinensis* with *Daemonorops draco* demonstrates enhanced activity maintenance.

Our findings highlight *Camellia sinensis* as a key facilitator in maintaining antioxidant activity, while singular extract compounds with dragon’s blood exhibit optimal activity. Intriguingly, compounding numerous extracts tends to diminish antioxidant activity, yielding an adverse effect.

This research significantly advances our scientific comprehension of the antioxidant properties inherent in these selected plants, promising avenues for leveraging their potential to augment the value of dragon’s blood in skincare and cosmetics. Moreover, the exploration of these natural plant extracts holds promise for revitalizing local agriculture. Thus, this study carries implications not only for the cosmetics industry but also for promoting sustainable agricultural practices.

## Figures and Tables

**Table 1:** Total anthocyanins and antioxidant capabilities of mixed natural plants using an ethanol conditioning mix.

	TAC (mg CGE/100 g)	TPC (mg GAE/100 g)	ABTS (%)	FRAP (mg/mL)
CS + PA	95.46 ± 14.99	9378.66 ± 27.69	92.70 ± 0.18	3.81 ± 0.02
CS + H1	59.50 ± 4.20	10,090.21 ± 386.48	89.31 ± 0.08	3.66 ± 0.08
HS + CT + PA	1926.94 ± 44.54	3764.77 ± 51.34	90.60 ± 0.67	2.33 ± 0.06
CT + CS + PA	211.46 ± 9.52	8016.33 ± 143.51	91.39 ± 0.72	3.67 ± 0.10
CS + PA + H3	30.45 ± 1.23	7672.55 ± 102.88	86.23 ± 0.14	3.63 ± 0.07
HS + CS + PA + H3	1199.37 ± 12.90	7300.55 ± 113.98	88.53 ± 0.02	3.72 ± 0.02
CT + CS + PA + H1	127.86 ± 4.30	6595.21 ± 145.75	89.17 ± 0.06	3.56 ± 0.06
HS + CT + CS + PA + H3	1037.33 ± 25.94	6308.33 ± 161.07	88.29 ± 0.29	3.61 ± 0.18
CS + DD	29.47 ± 1.38	9718.99 ± 86.90	93.29 ± 0.08	3.71 ± 0.07
CS + PA + DD	25.36 ± 4.39	7466.99 ± 71.99	92.95 ± 0.07	3.71 ± 0.08
CS + H1 + DD	3.11 ± 4.2	7366.88 ± 83.77	91.98 ± 0.19	3.69 ± 0.01
HS + CT + PA + DD	991.33 ± 24.22	2967.21 ± 37.18	92.27 ± 0.13	1.83 ± 0.03
CT + CS + PA + DD	131.92 ± 9.63	6597.99 ± 51.51	92.71 ± 0.18	3.58 ± 0.03
CS + PA + H3 + DD	29.56 ± 3.58	6494.99 ± 22.28	90.06 ± 0.04	3.66 ± 0.08
HS + CS + PA + H3 + DD	822.48 ± 10.83	5940.44 ± 110.90	90.20 ± 0.08	3.54 ± 0.09
CT + CS + PA + H1 + DD	68.31 ± 4.69	6279.10 ± 124.99	91.57 ± 0.12	3.44 ± 0.13
HS + CT + CS + PA + H3 + DD	656.75 ± 19.27	5809.99 ± 36.76	89.43 ± 0.20	3.51 ± 0.04
CS + DC	104.13 ± 47.04	10,500.21 ± 122.99	92.20 ± 0.27	3.73 ± 0.06
CS + PA + DC	126.30 ± 36.46	8333.99 ± 33.93	88.87 ± 2.12	3.68 ± 0.10
CS + H1 + DC	77.26 ± 23.70	8218.55 ± 58.78	91.41 ± 0.33	3.68 ± 0.10
HS + CT + PA + DC	1075.58 ± 30.59	3723.66 ± 63.38	89.87 ± 0.26	2.02 ± 0.05
CT + CS + PA + DC	303.63 ± 23.36	6712.55 ± 1.9.67	91.01 ± 0.12	3.61 ± 0.15
CS + PA + H3 + DC	52.43 ± 1.20	6606.77 ± 127.32	87.63 ± 0.26	3.51 ± 0.15
HS + CS + PA + H3 + DC	799.32 ± 28.65	6248.44 ± 127.12	86.69 ± 0.23	3.54 ± 0.12
CT + CS + PA + H1 + DC	112.48 ± 3.79	6308.10 ± 73.93	86.84 ± 0.27	3.57 ± 0.10
HS + CT + CS + PA + H3 + DC	733.53 ± 34.69	6052.21 ± 143.25	86.68 ± 0.27	3.59 ± 0.22

Abbreviations: HS, *Hibiscus sabdariffa*, Roselle; CT, *Clitoria ternatea*, butterfly pea flower; GE, commercial green tea extract; CS, *Camellia sinensis*, green tea; PA, *Pandanus amaryllifolius*, pandan leaves; H1, dry *Hylocereus* sp., red dragon’s fruit flowers (dry); H2, dry *Hylocereus* sp. petals, red dragon’s fruit flower petals (dry); H3, dry *Hylocereus* sp. stamens, red dragon’s fruit flower stamens (dry); H4, wet *Hylocereus* sp. petals, red dragon’s fruit flower petals (wet); H5, wet *Hylocereus* sp. stamens, red dragon’s fruit flower stamens (wet); DD, *Daemonorops draco;* DC, *Dracaena cochinchinensis*. Results show the mean ± standard deviation (n = 3).

## Data Availability

Data and materials are available on reasonable request by email to the corresponding author.

## Supplementary Materials

The following supporting information can be downloaded at: https://www.mdpi.com/article/10.3390/life14070786/s1.
