# Supplementary material for: Combining Different Natural Plant Extracts to Stabilize the Antioxidative Activity of Dragon’s Blood
Source: Life (Basel). 2024 Jun 21;14(7):786. doi: 10.3390/life14070786 (PMC11277873; doi:10.3390/life14070786)
Supplement: Supplementary file 1 [file life-14-00786-s001.zip › life-2999011-supplementary.pdf]

# Supplementary data 1

List S1: Commercial Dragon's Blood name and their sample
No.: Jinji brand (*Dracaena* 1)
Jinxing brand (*Dracaena* 2)
Baozhu brand (*Dracaena* 3)
Crown brand (*Dracaena* 4)
Xianfeng brand (*Dracaena* 5)
Hong Kong orchid brand (*Dracaena* 6)
Baozhu brand Qilin brand (*Dracaena* 7)
Chungyaon Qilin Dragon's Blood (*Dracaena* 8)
Draco Dragon's Blood (*Dracaena* 9)

Figures are now shown in the main text.

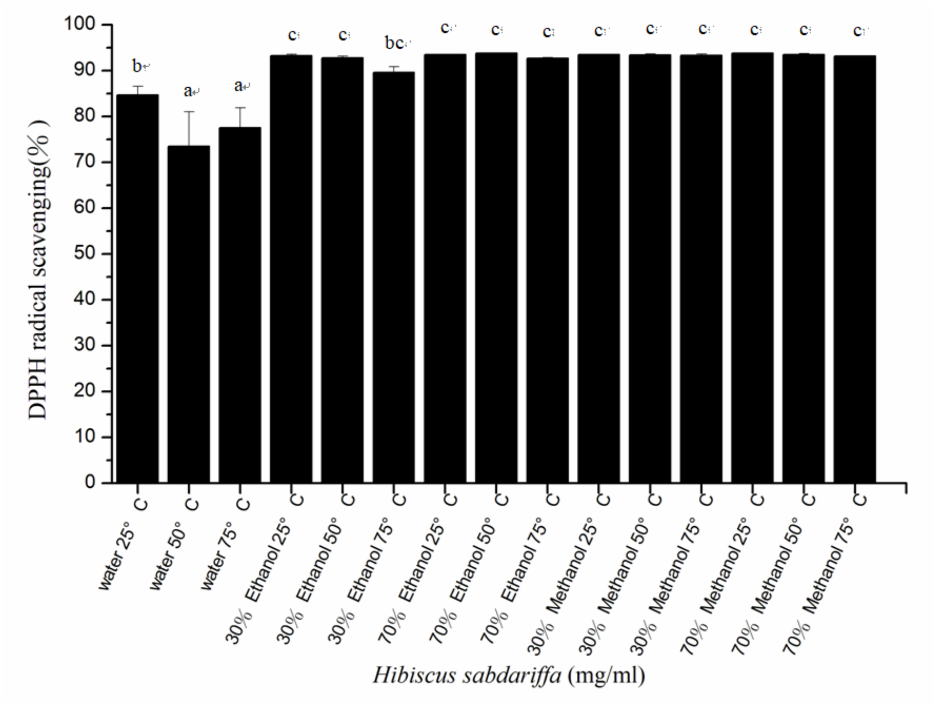


**Figure S1.** Performance of *Hibiscus sabdariff* in DPPH under different extraction conditions**.** a-c Means ($n$ = 3) with different superscripts in a column of separate sample are significantly different ($p < 0.05$).

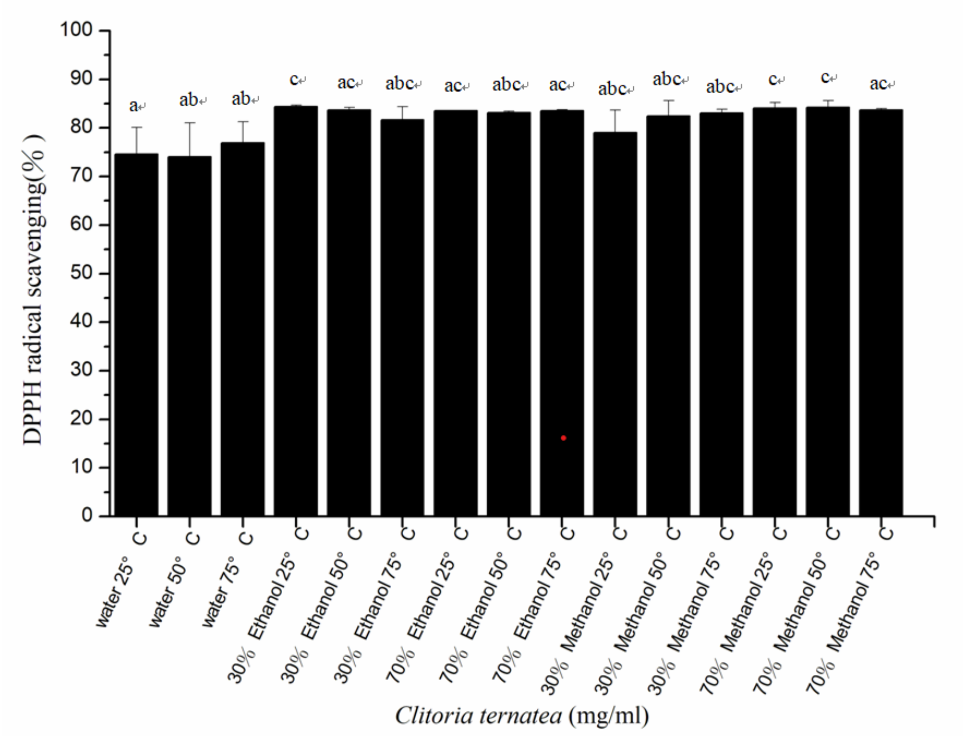


**Figure S2.** Performance of *Clitoria ternatea* in DPPH under different extraction conditions. a-c Means ($n$ = 3) with different superscripts in a column of separate sample are significantly different ($p < 0.05$).

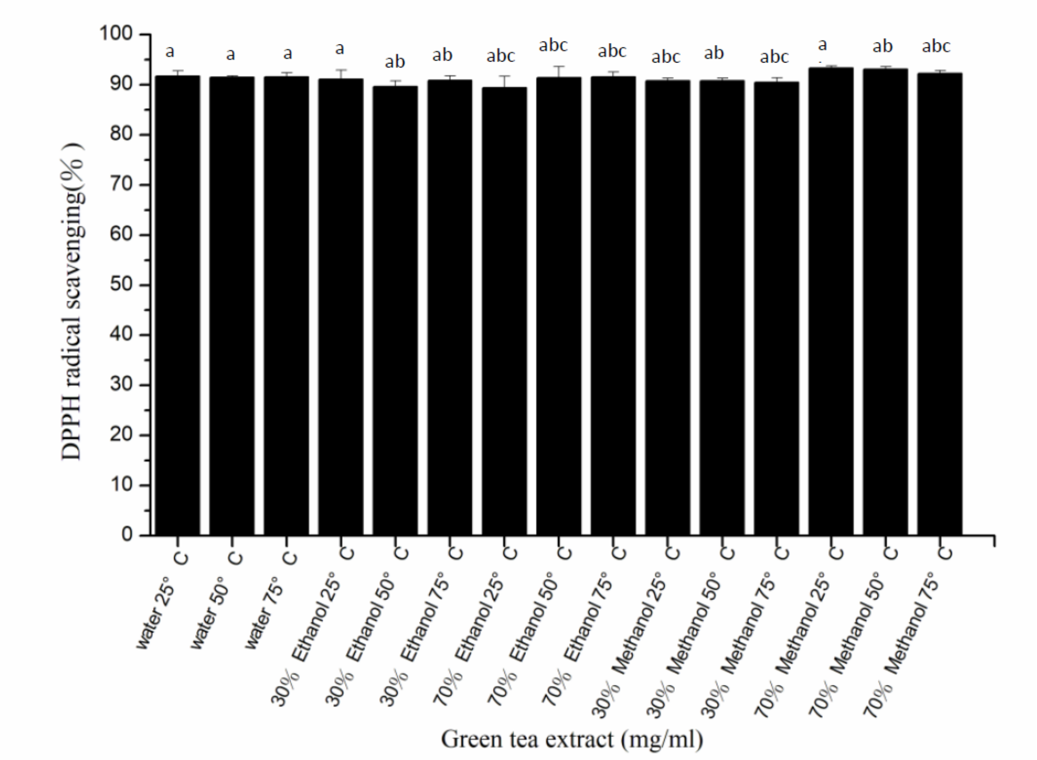


**Figure S3.** Performance of Green tea extract in DPPH under different extraction conditions. a-c Means ($n = 3$) with different superscripts in a column of separate sample are significantly different ($p < 0.05$).

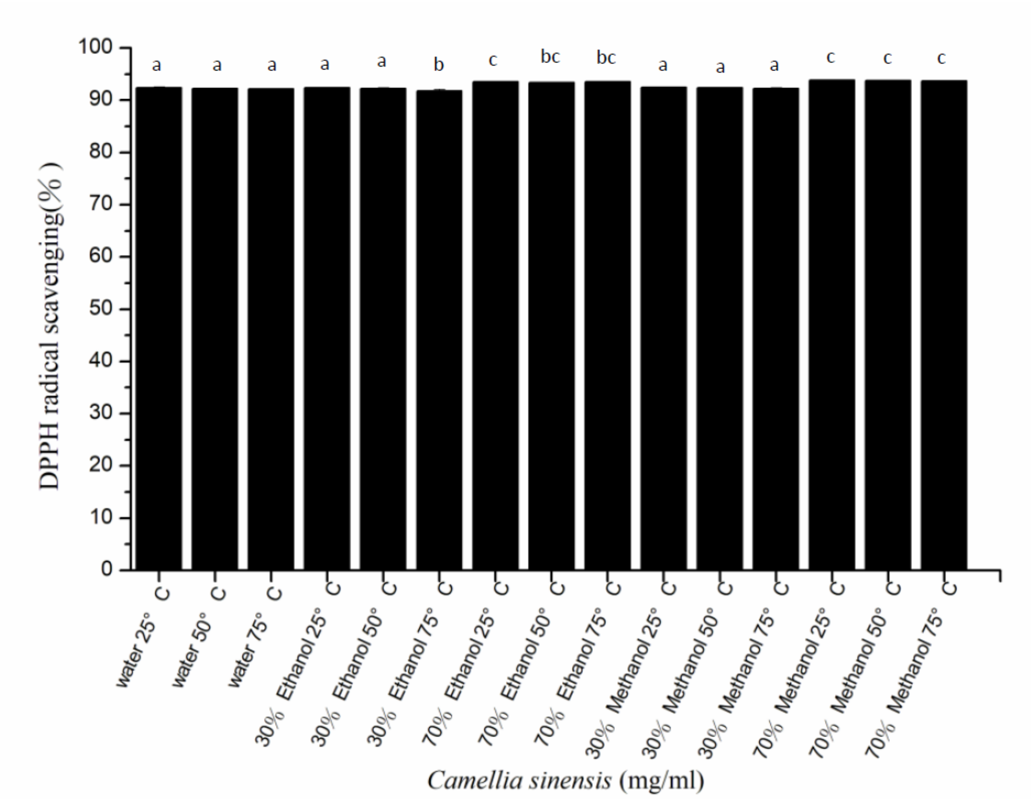


**Figure S4.** Performance of *Camellia sinensis* in DPPH under different extraction conditions. a-c Means ($n = 3$) with different superscripts in a column of separate sample are significantly different ($p < 0.05$).

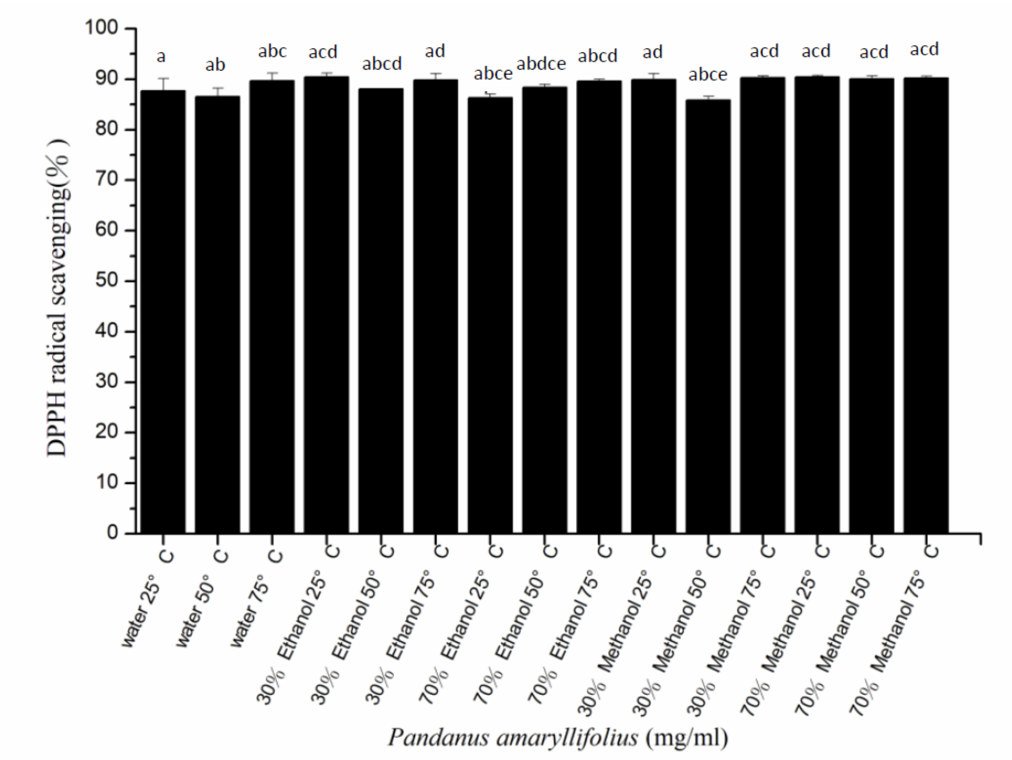


**Figure S5.** Performance of *Pandanus amaryllifolius* in DPPH under different extraction conditions. a-e Means ($n = 3$) with different superscripts in a column of separate sample are significantly different ($p < 0.05$).

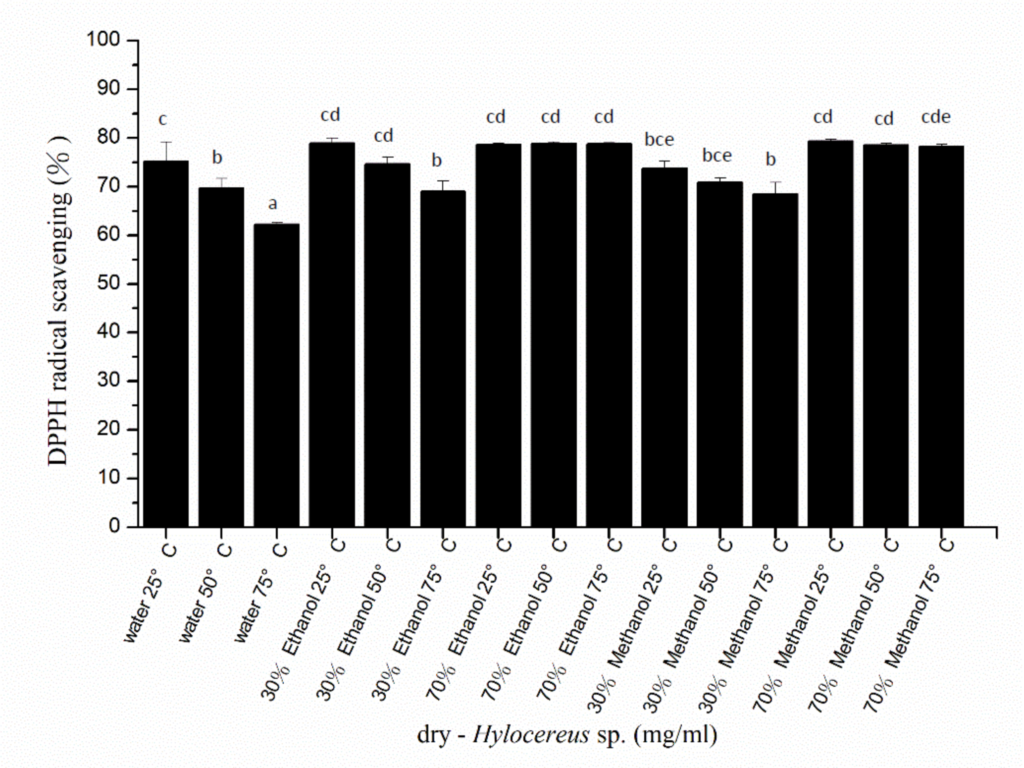


**Figure S6.** Performance of dry - *Hylocereus* sp. in DPPH under different extraction conditions. a-e Means ($n = 3$) with different superscripts in a column of separate sample are significantly different ($p < 0.05$).

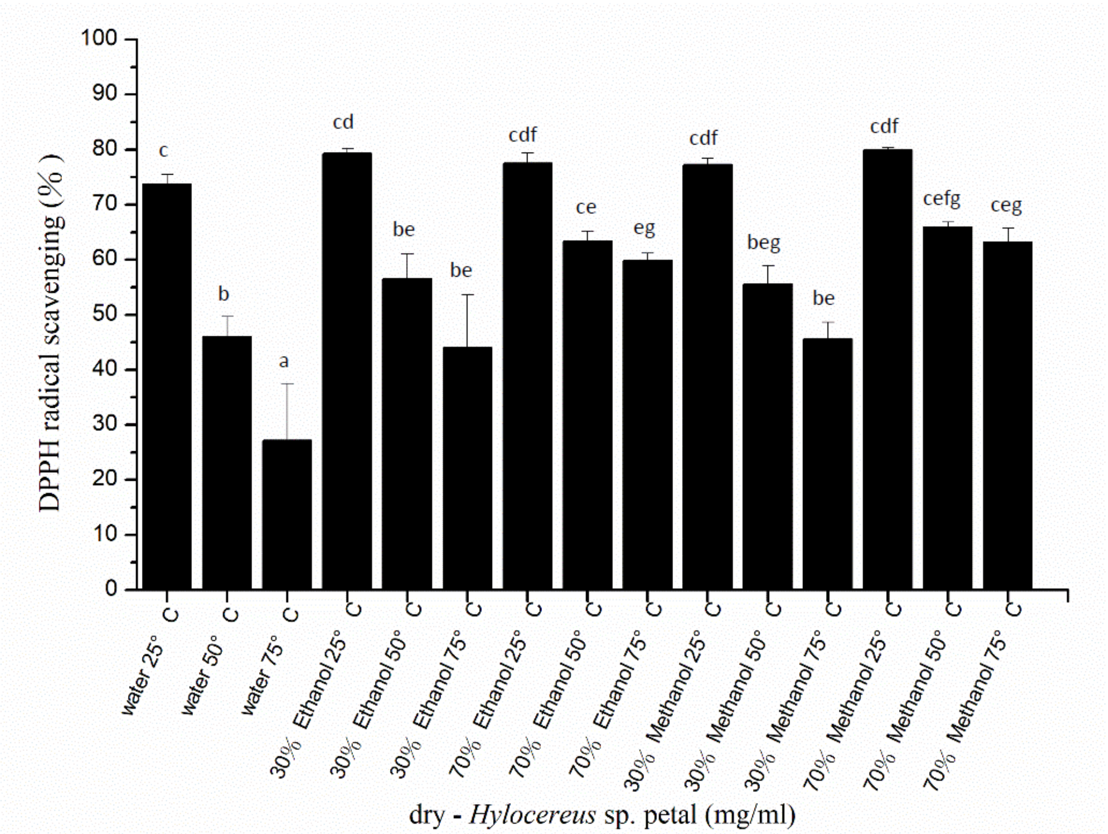


**Figure S7.** Performance of dry - *Hylocereus* sp. petal in DPPH under different extraction conditions. a-g Means ($n$ = 3) with different superscripts in a column of separate sample are significantly different ($p < 0.05$).

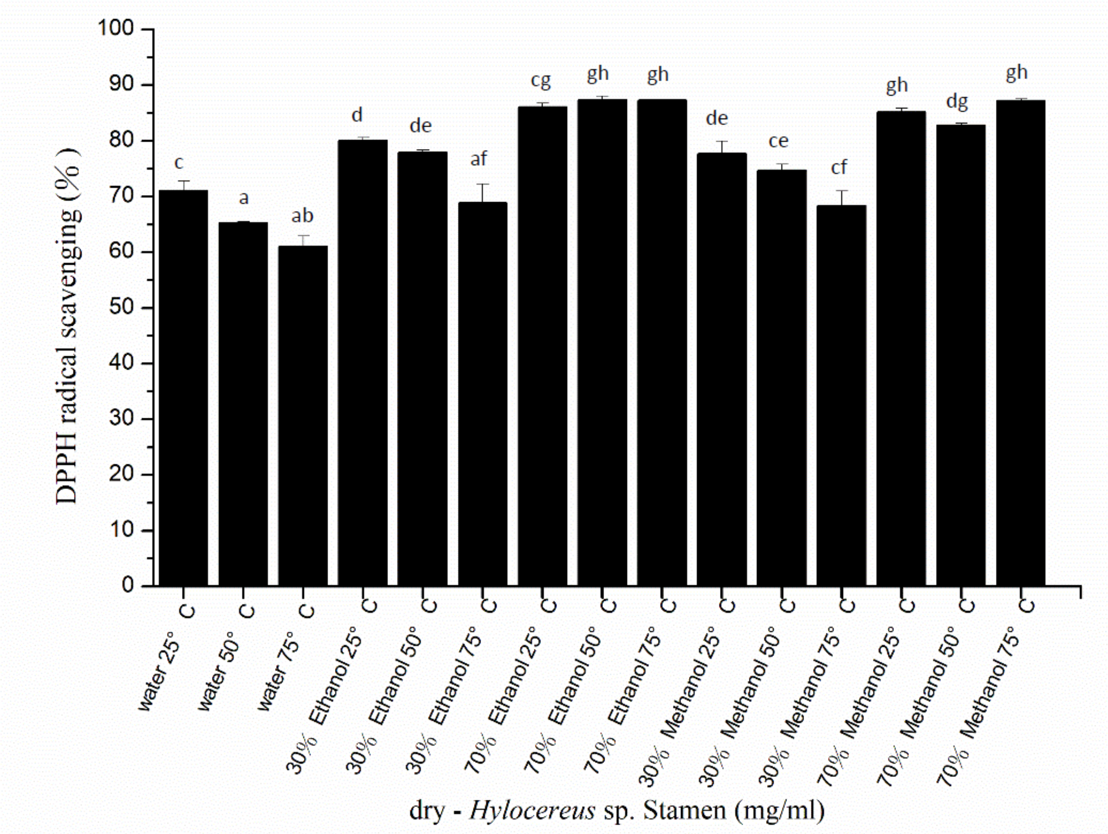

**Figure S8.** Performance of dry - *Hylocereus* sp. Stamen in DPPH under different extraction conditions. a-h Means ($n = 3$) with different superscripts in a column of separate sample are significantly different ($p < 0.05$).

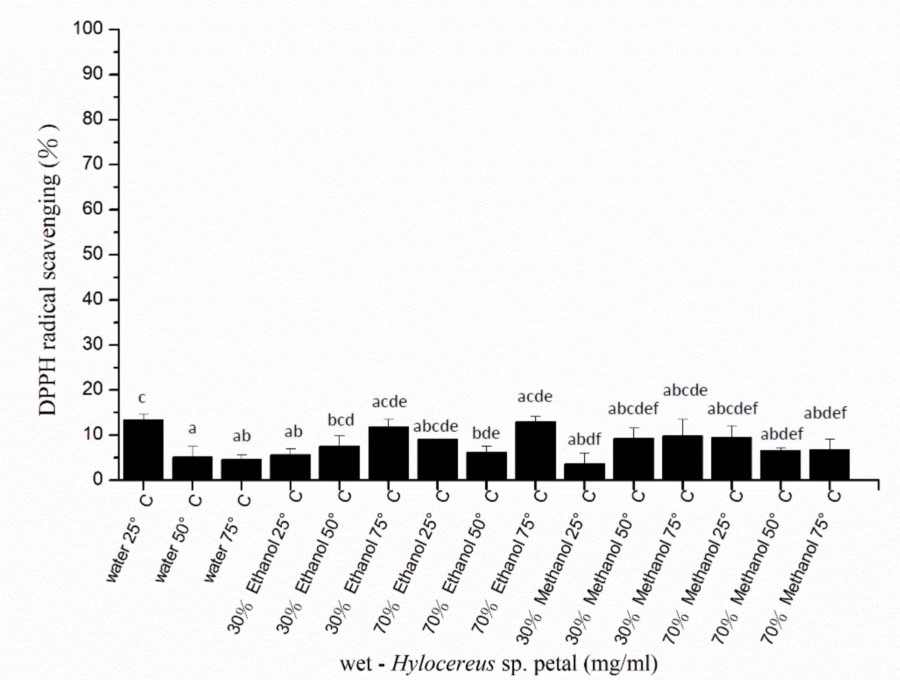


**Figure S9.** Performance of wet - *Hylocereus* sp. petal in DPPH under different extraction conditions. a-f Means ($n = 3$) with different superscripts in a column of separate sample are significantly different ($p < 0.05$).

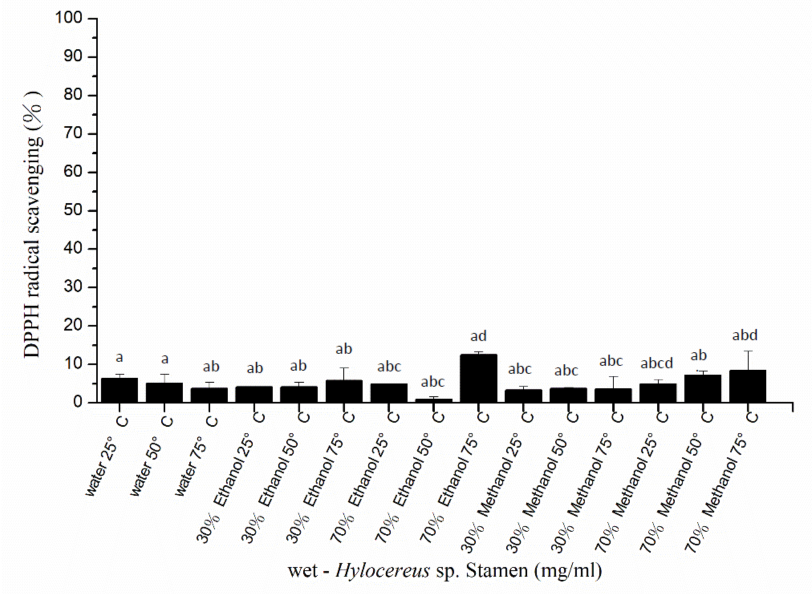


**Figure S10.** Performance of wet - *Hylocereus* sp. Stamenin DPPH under different extraction conditions. a-d Means ($n = 3$) with different superscripts in a column of separate sample are significantly different ($p < 0.05$).

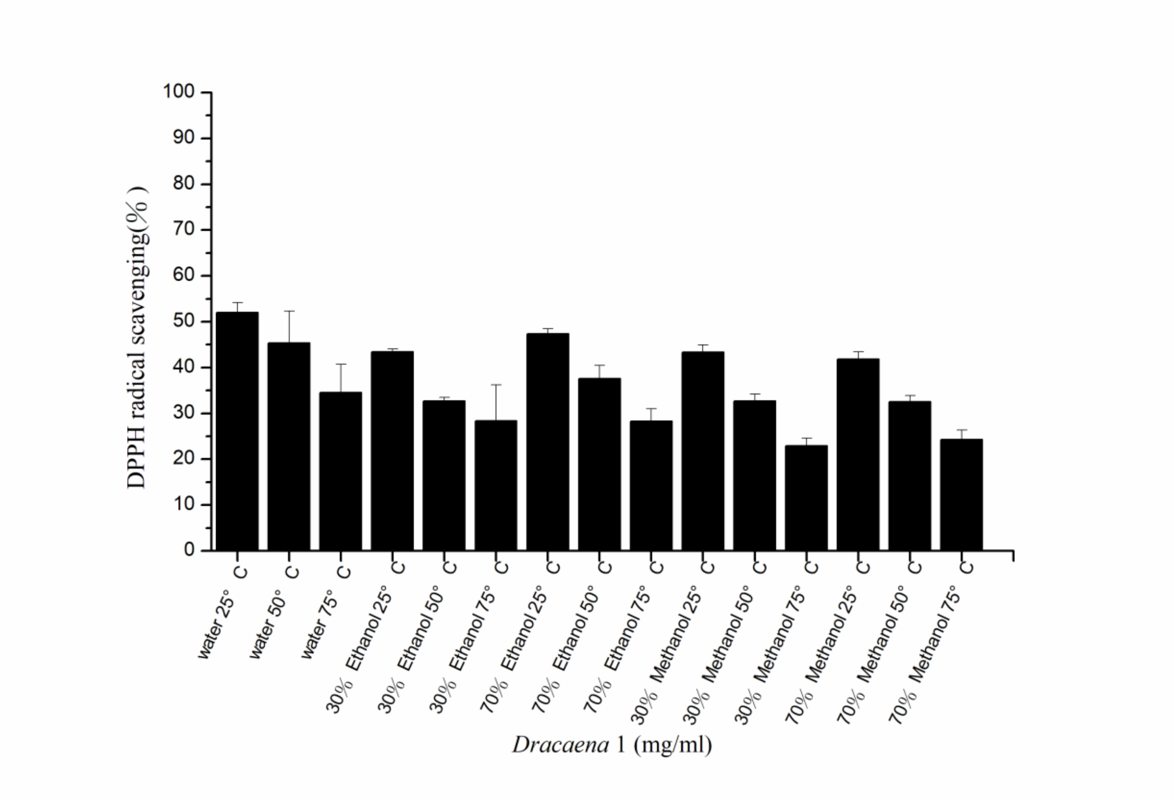


**Figure S11.** Performance of *Dracaena* 1 in DPPH under different extraction conditions. a-h Means ($n$ = 3) with different superscripts in a column of separate sample are significantly different ($p < 0.05$).

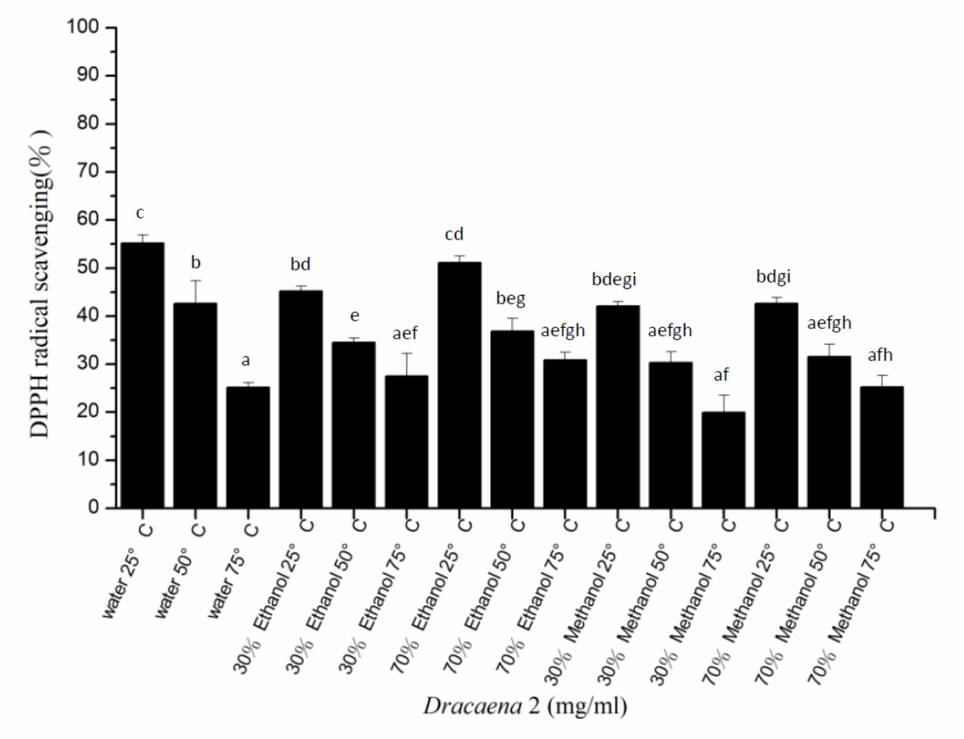


**Figure S12.** Performance of *Dracaena* 2 in DPPH under different extraction conditions. a-i Means ($n$ = 3) different superscripts in a column of separate sample are significantly different ($p < 0.05$).

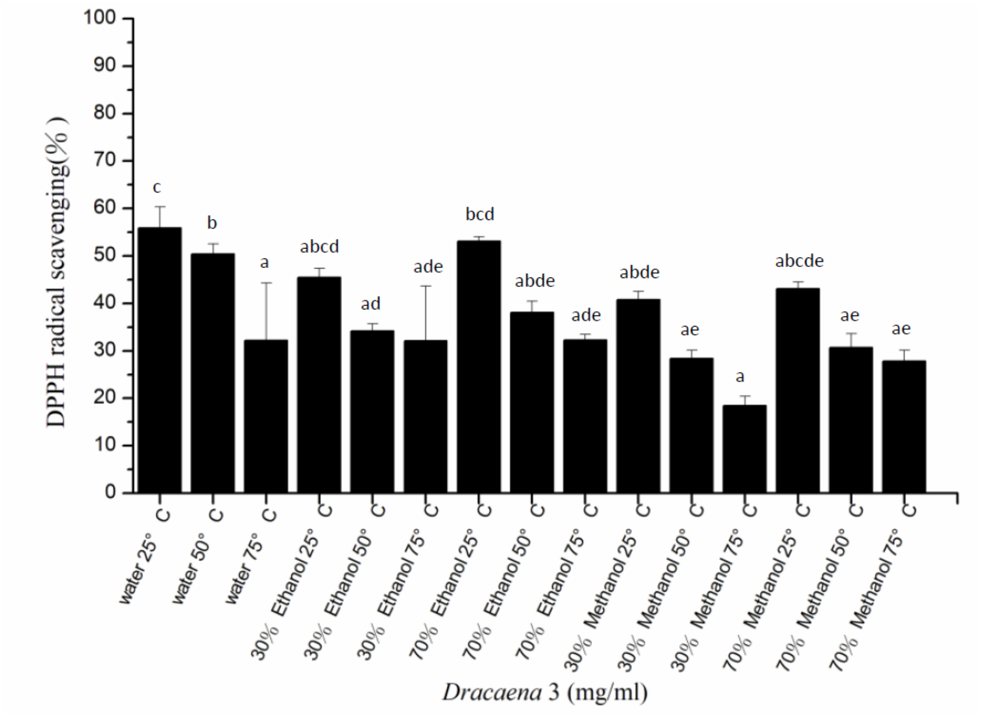


**Figure S13.** Performance of *Dracaena* 3 in DPPH under different extraction conditions. a-e Means (*n* = 3) ）with different superscripts in a column of separate sample are significantly different ($p < 0.05$).

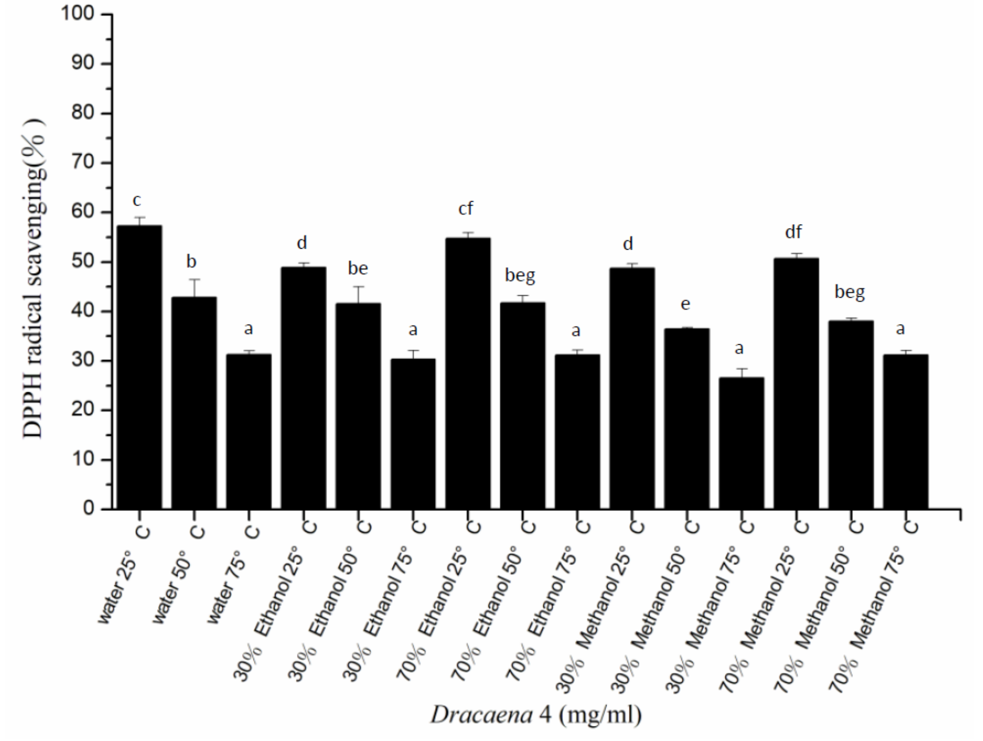


**Figure S14.** Performance of *Dracaena* 4 in DPPH under different extraction conditions. a-g Means (*n* = 3) with different superscripts in a column of separate sample are significantly different ($p < 0.05$).

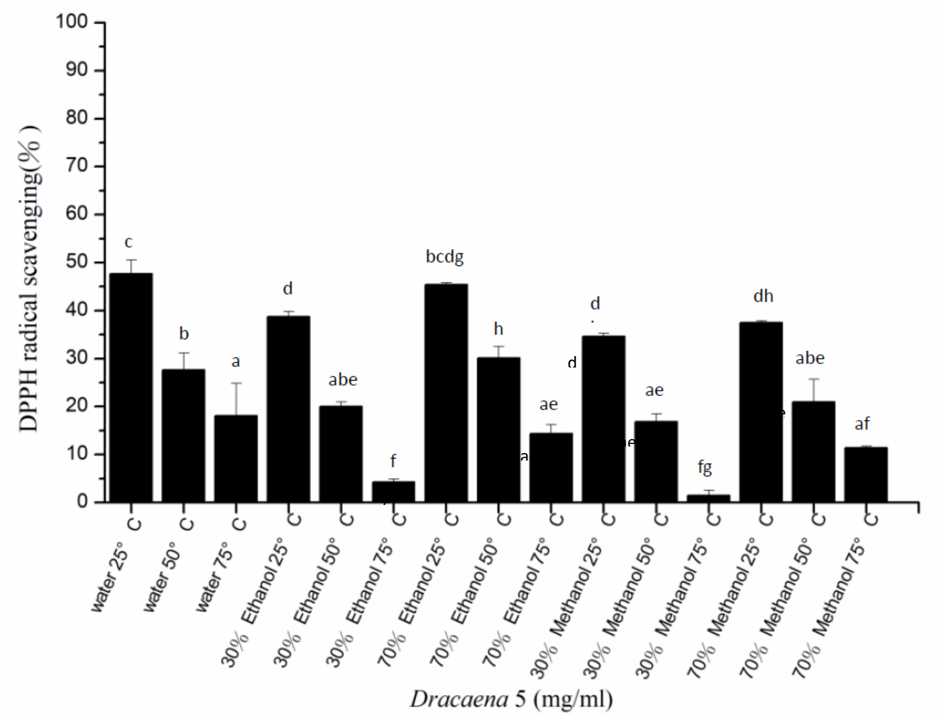


**Figure S15.** Performance of *Dracaena* 5 in DPPH under different extraction conditions. a-h Means ($n$ = 3) with different superscripts in a column of separate sample are significantly different ($p < 0.05$).

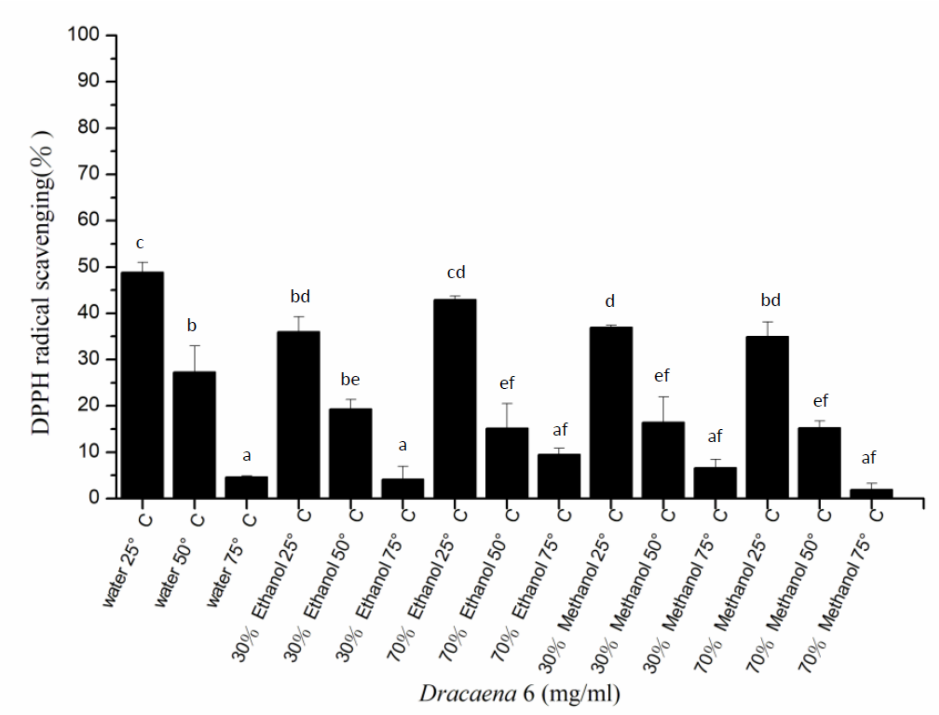


**Figure S16.** Performance of *Dracaena* 6 in DPPH under different extraction conditions. a-f Means ($n$ = 3) with different superscripts in a column of separate sample are significantly different ($p < 0.05$).

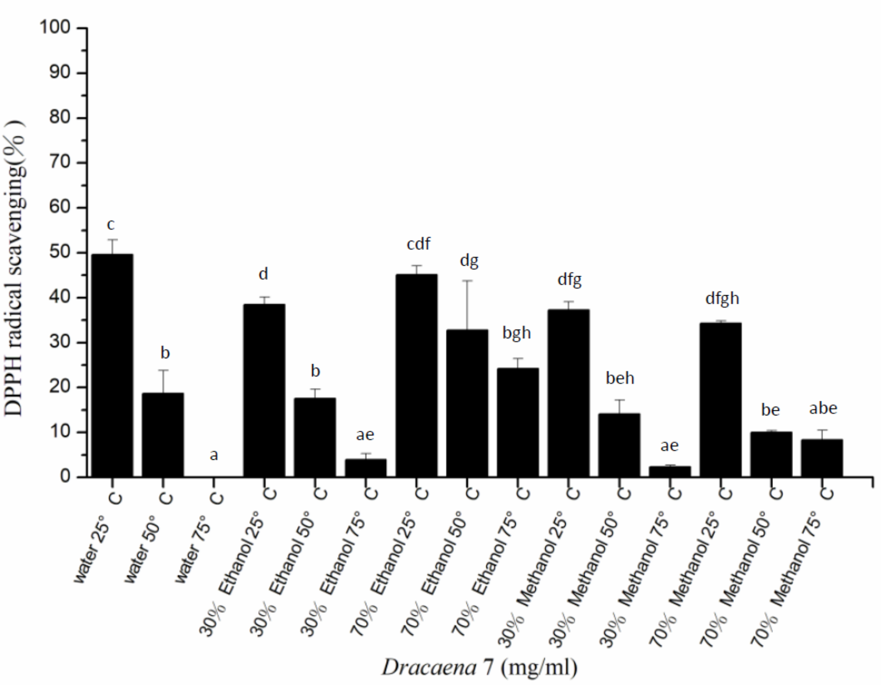


**Figure S17.** Performance of *Dracaena* 7 in DPPH under different extraction conditions. a-h Means ($n$ = 3) with different superscripts in a column of separate sample are significantly different ($p < 0.05$).

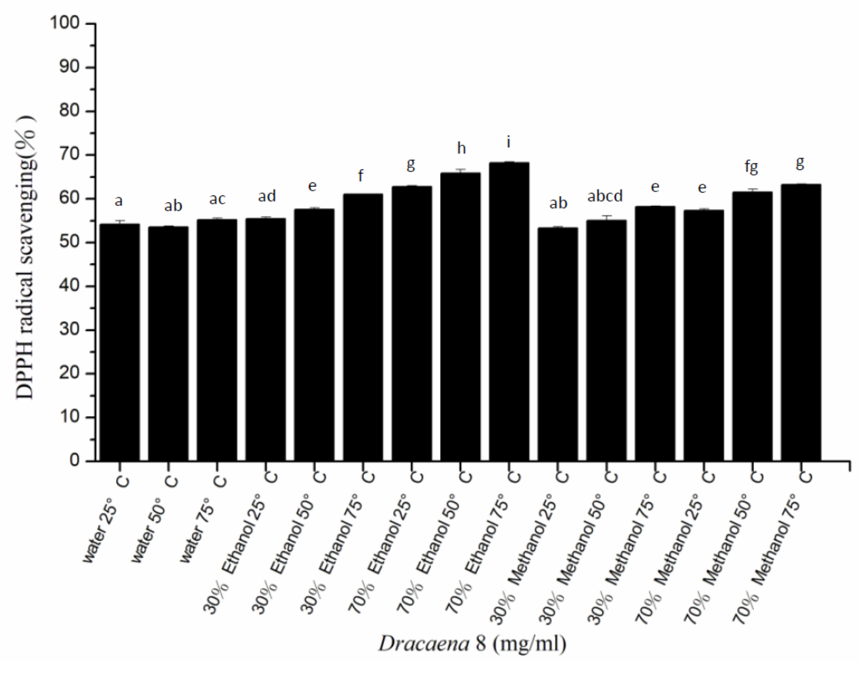


**Figure S18.** Performance of *Dracaena* 8 in DPPH under different extraction conditions. a-i Means ($n$ = 3) with different superscripts in a column of separate sample are significantly different ($p < 0.05$).

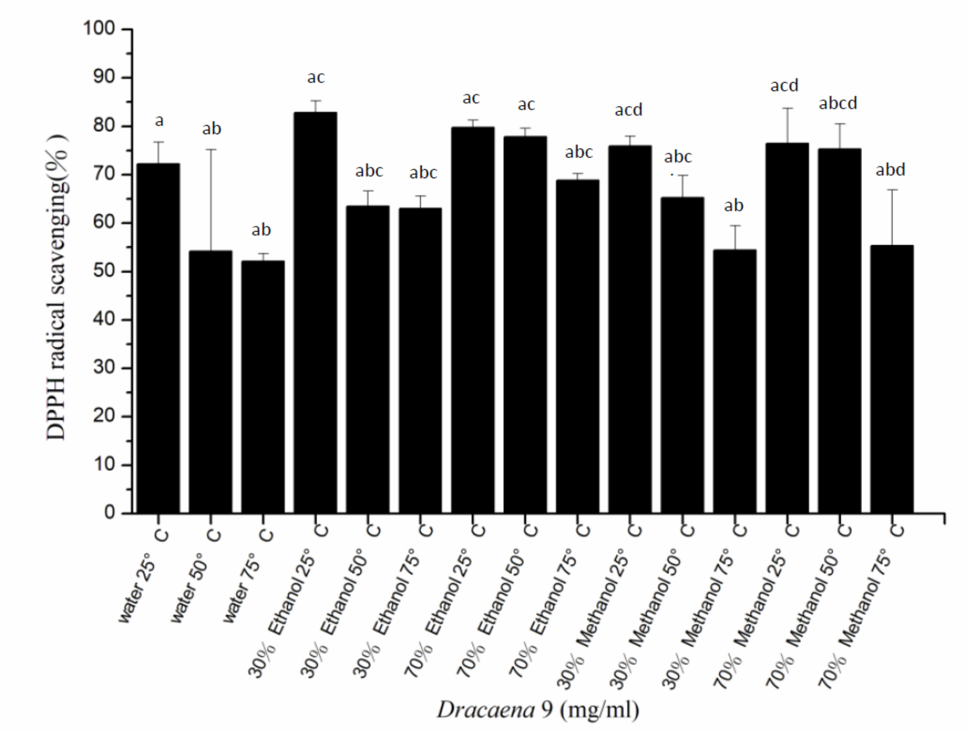


**Figure S19.** Performance of *Dracaena* 9 in DPPH under different extraction conditions. a-d Means ($n$ = 3) with different superscripts in a column of separate sample are significantly different ($p < 0.05$).

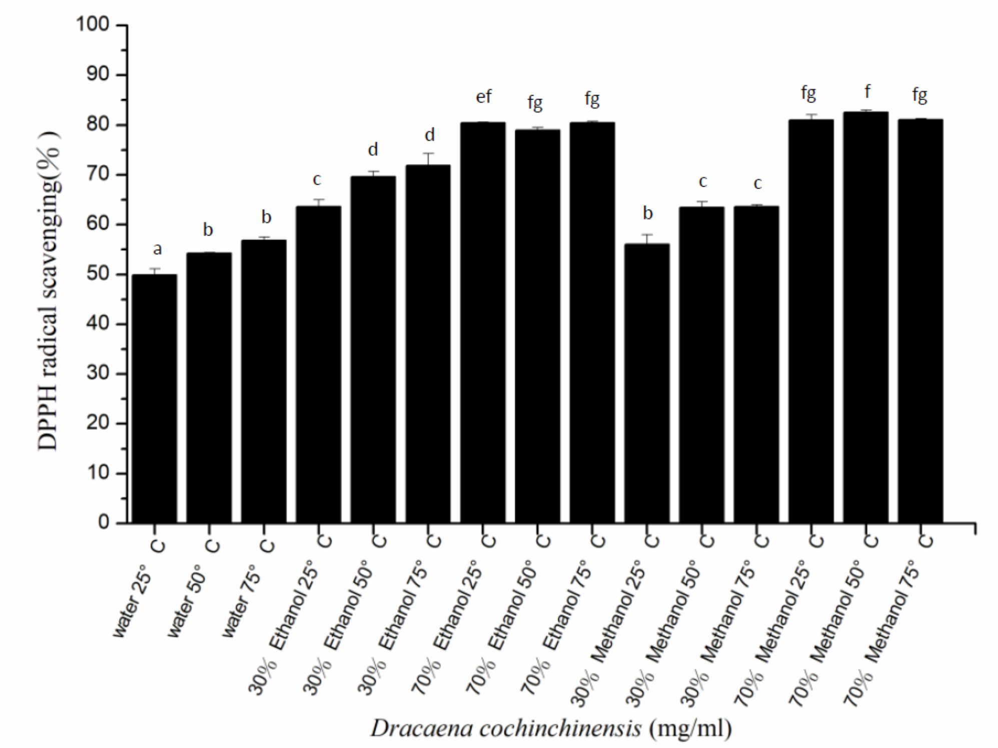


**Figure S20.** Performance of *Dracaena cochinchinensis* in DPPH under different extraction conditions. a-g Means ($n$ = 3) with different superscripts in a column of separate sample are significantly different ($p$ < 0.05).

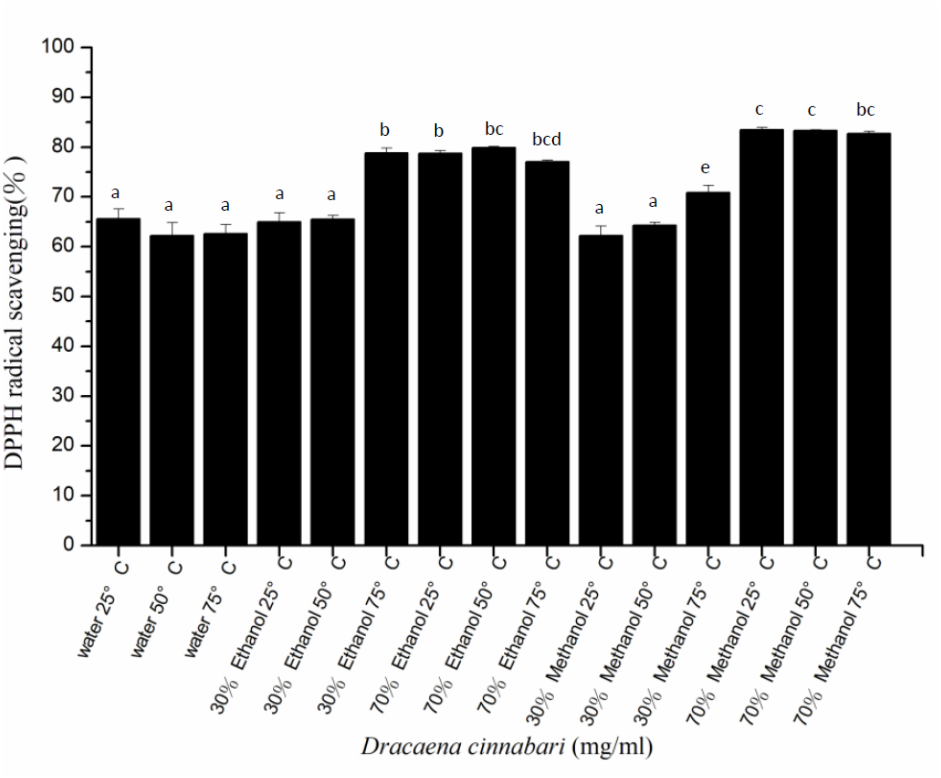


**Figure S21.** Performance of *Dracaena cinnabari* in DPPH under different extraction conditions. a-e Means ($n$ = 3) with different superscripts in a column of separate sample are significantly different ($p < 0.05$).

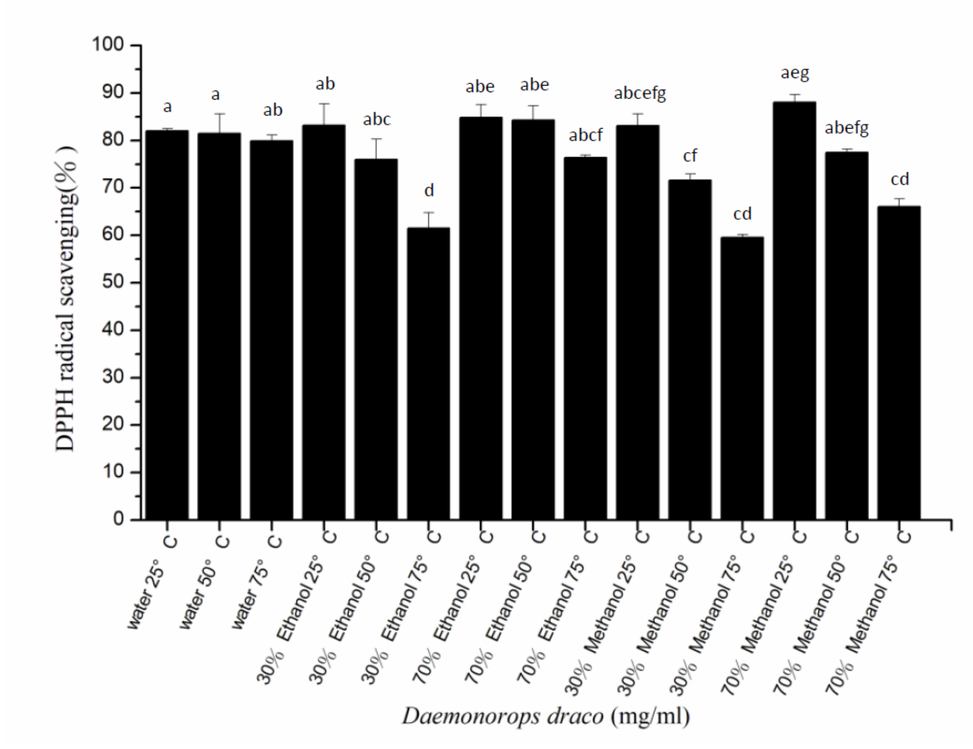


**Figure S22.** Performance of *Daemonorops draco* in DPPH under different extraction conditions. a-g Means ($n$ = 3) with different superscripts in a column of separate sample are significantly different ($p < 0.05$).

# Supplementary data 2

Tables are now shown in the main text.

**Table S2.** Total anthocyanins and antioxidant capacity of *Hibiscus sabdariffa* under different extraction conditions.

| | | TAC (mg CGE/100g) | TPC (mg GAE/100g) | ABTS (%) | FRAP (mg/mL) |
|---|---|---|---|---|---|
| water | 25°C | 3947.28 ± 62.00 | 3562.44 ± 13.73 | 98.11 ± 0.06 | 2.96 ± 0.04 |
| | 50°C | 4236.67 ± 18.33 | 3599.99 ± 52.87 | 97.84 ± 0.22 | 2.99 ± 0.02 |
| | 75°C | 3939.99 ± 69.02 | 3529.66 ± 44.51 | 98.08 ± 0.03 | 2.69 ± 0.03 |
| 30% Ethanol | 25°C | 3717.56 ± 50.44 | 3210.21 ± 43.50 | 98.11 ± 0.10 | 2.97 ± 0.04 |
| | 50°C | 4084.99 ± 44.09 | 3743.77 ± 54.46 | 97.97 ± 0.14 | 3.10 ± 0.04 |
| | 75°C | 3646.54 ± 54.88 | 3710.77 ± 74.72 | 97.83 ± 0.04 | 3.27 ± 0.06 |
| 70% Ethanol | 25°C | 3432.46 ± 37.33 | 2986.66 ± 39.57 | 98.00 ± 0.06 | 3.00 ± 0.02 |
| | 50°C | 3318.68 ± 61.29 | 3181.77 ± 18.51 | 98.01 ± 0.04 | 3.05 ± 0.04 |
| | 75°C | 4002.11 ± 77.10 | 3678.66 ± 33.84 | 97.59 ± 0.13 | 3.23 ± 0.10 |
| 30% Methanol | 25°C | 3669.64 ± 82.94 | 3275.99 ± 37.49 | 98.09 ± 0.05 | 3.00 ± 0.01 |
| | 50°C | 3951.35 ± 122.12 | 3618.77 ± 39.99 | 98.02 ± 0.04 | 3.13 ± 0.06 |
| | 75°C | 3745.06 ± 36.41 | 3553.88 ± 13.73 | 98.05 ± 0.01 | 2.49 ± 0.04 |
| 70% Methanol | 25°C | 4266.12 ± 66.95 | 3618.10 ± 27.84 | 97.86 ± 0.02 | 2.64 ± 0.02 |
| | 50°C | 4030.28 ± 161.32 | 3562.44 ± 34.50 | 97.85 ± 0.05 | 2.94 ± 0.08 |
| | 75°C | 4025.32 ± 71.86 | 3568.10 ± 19.59 | 97.68 ± 0.04 | 3.18 ± 0.02 |

a-g Means ($n$ = 3) with different superscripts in a column of separate sample are significantly different ($p < 0.05$).

**Table S3.** Total anthocyanins and antioxidant capacity of *Clitoria ternatea* under different extraction conditions.

| | | TAC (mg CGE/100g) | TPC (mg GAE/100g) | ABTS (%) | FRAP (mg/mL) |
|---|---|---|---|---|---|
| water | 25°C | 170.33 ± 9.86 | 3176.77 ± 15.570 | 97.34 ± 0.23 | 1.93 ± 0.03 |
| | 50°C | 205.28 ± 14.70 | 3030.55 ± 21.64 | 97.50 ± 0.39 | 2.03 ± 0.02 |
| | 75°C | 187.42 ± 11.18 | 3047.99 ± 48.06 | 97.81 ± 0.21 | 1.98 ± 0.04 |
| 30% Ethanol | 25°C | 238.13 ± 9.40 | 2951.88 ± 22.63 | 97.64 ± 0.52 | 1.74 ± 0.01 |
| | 50°C | 220.76 ± 14.67 | 2935.66 ± 35.45 | 97.95 ± 0.02 | 2.09 ± 0.05 |
| | 75°C | 175.12 ± 10.74 | 2964.21 ± 47.53 | 97.77 ± 0.30 | 2.02 ± 0.05 |
| 70% Ethanol | 25°C | 103.03 ± 10.99 | 2554.55 ± 36.40 | 97.80 ± 0.03 | 1.56 ± 0.01 |
| | 50°C | 123.07 ± 6.06 | 2826.10 ± 45.77 | 97.78 ± 0.04 | 1.73 ± 0.04 |
| | 75°C | 144.83 ± 9.86 | 2641.77 ± 48.75 | 97.74 ± 0.06 | 1.66 ± 0.07 |
| 30% Methanol | 25°C | 229.16 ± 16.25 | 3067.55 ± 23.10 | 96.24 ± 0.55 | 1.87 ± 0.01 |
| | 50°C | 244.97 ± 2.03 | 2973.66 ± 11.57 | 97.59 ± 0.54 | 2.00 ± 0.02 |
| | 75°C | 205.23 ± 0.93 | 3079.33 ± 13.00 | 97.69 ± 0.46 | 1.86 ± 0.03 |
| 70% Methanol | 25°C | 155.41 ± 12.76 | 2662.10 ± 41.43 | 97.79 ± 0.07 | 1.80 ± 0.01 |
| | 50°C | 135.98 ± 6.30 | 2759.10 ± 18.70 | 97.74 ± 0.04 | 1.79 ± 0.02 |
| | 75°C | 178.68 ± 4.76 | 2688.99 ± 11.50 | 97.66 ± 0.26 | 2.07 ± 0.04 |

a-i Means ($n$ = 3) with different superscripts in a column of separate sample are significantly different ($p < 0.05$).

**Table S4.** Total anthocyanins and antioxidant capacity of a commercial green tea extract under different extraction conditions.

| | | TAC (mg CGE/100g) | TPC (mg GAE/100g) | ABTS (%) | FRAP (mg/mL) |
|---|---|---|---|---|---|
| water | 25°C | - | 522.10 ± 28.74 | 98.38 ± 0.03 | 1.15 ± 0.03 |
| | 50°C | - | 668.10 ± 3.66 | 98.35 ± 0.05 | 1.26 ± 0.02 |
| | 75°C | - | 707.44 ± 3.66 | 98.35 ± 0.03 | 1.29 ± 0.01 |
| 30% Ethanol | 25°C | - | 692.44 ± 9.25 | 98.33 ± 0.02 | 1.27 ± 0.01 |
| | 50°C | - | 748.33 ± 3.84 | 98.39 ± 0.02 | 1.39 ± 0.02 |
| | 75°C | - | 740.33 ± 11.02 | 98.39 ± 0.04 | 1.23 ± 0.02 |
| 70% Ethanol | 25°C | - | 756.44 ± 12.92 | 98.37 ± 0.02 | 1.52 ± 0.02 |
| | 50°C | - | 637.99 ± 5.90 | 98.36 ± 0.01 | 1.23 ± 0.02 |
| | 75°C | - | 720.88 ± 9.35 | 98.27 ± 0.04 | 1.22 ± 0.02 |
| 30% Methanol | 25°C | - | 640.55 ± 0.51 | 98.36 ± 0.04 | 1.33 ± 0.01 |
| | 50°C | - | 636.55 ± 1.26 | 98.37 ± 0.02 | 1.30 ± 0.01 |
| | 75°C | - | 663.10 ± 4.35 | 98.31 ± 0.03 | 1.45 ± 0.01 |
| 70% Methanol | 25°C | - | 714.77 ± 5.40 | 98.27 ± 0.03 | 1.19 ± 0.02 |
| | 50°C | - | 505.33 ± 4.84 | 98.29 ± 0.05 | 1.15 ± 0.03 |
| | 75°C | - | 727.66 ± 1.53 | 98.33 ± 0.07 | 1.35 ± 0.01 |

a-i Means ($n$ = 3) with different superscripts in a column of separate sample are significantly different ($p < 0.05$).

**Table S5.** Total anthocyanins and antioxidant capacity of *Camellia sinensis* (green tea leaves) under different extraction condition.s

| | | TAC (mg CGE/100g) | TPC (mg GAE/100g) | ABTS (%) | FRAP (mg/mL) |
|---|---|---|---|---|---|
| water | 25°C | - | 9073.88 ± 43.91 | 98.29 ± 0.07 | 3.82 ± 0.02 |
| | 50°C | - | 9308.88 ± 142.26 | 98.28 ± 0.01 | 3.76 ± 0.08 |
| | 75°C | - | 9255.10 ± 85.75 | 98.26 ± 0.06 | 3.82 ± 0.03 |
| 30% Ethanol | 25°C | 18.54 ± 0.44 | 9684.99 ± 122.90 | 98.30 ± 0.04 | 3.82 ± 0.05 |
| | 50°C | 56.33 ± 7.05 | 9696.99 ± 204.87 | 98.20 ± 0.01 | 3.73 ± 0.09 |
| | 75°C | 68.24 ± 3.24 | 9921.66 ± 225.83 | 98.05 ± 0.03 | 3.76 ± 0.08 |
| 70% Ethanol | 25°C | 112.22 ± 6.14 | 9840.88 ± 117.38 | 98.25 ± 0.01 | 3.80 ± 0.05 |
| | 50°C | 217.42 ± 23.76 | 9637.21 ± 52.98 | 98.20 ± 0.01 | 3.74 ± 0.11 |
| | 75°C | 249.26 ± 27.82 | 9792.21 ± 63.92 | 97.39 ± 0.03 | 3.85 ± 0.00 |
| 30% Methanol | 25°C | 6.29 ± 2.59 | 9409.33 ± 67.45 | 98.35 ± 0.05 | 3.85 ± 0.00 |
| | 50°C | 25.60 ± 2.53 | 9731.44 ± 91.88 | 98.29 ± 0.04 | 3.70 ± 0.13 |
| | 75°C | 28.44 ± 1.95 | 9598.99 ± 126.73 | 98.22 ± 0.01 | 3.78 ± 0.11 |
| 70% Methanol | 25°C | 41.19 ± 3.53 | 9628.55 ± 91.35 | 98.28 ± 0.02 | 3.76 ± 0.14 |
| | 50°C | 26.22 ± 3.49 | 9048.88 ± 116.17 | 98.22 ± 0.01 | 3.73 ± 0.02 |
| | 75°C | 102.81 ± 9.60 | 9606.21 ± 132.50 | 98.04 ± 0.02 | 3.79 ± 0.09 |

a-g Means ($n$ = 3) with different superscripts in a column of separate sample are significantly different ($p < 0.05$).

**Table S6.** Total anthocyanins and antioxidant capacity of *Pandanus amaryllifolius* (pandan) under different extraction conditions.

| | | TAC (mg CGE/100g) | TPC (mg GAE/100g) | ABTS (%) | FRAP (mg/mL) |
|---|---|---|---|---|---|
| water | 25°C | - | 1628.88 ± 26.96 | 98.25 ± 0.04 | 0.87 ± 0.03 |
| | 50°C | - | 1897.21 ± 62.01 | 98.07 ± 0.36 | 0.93 ± 0.01 |
| | 75°C | - | 1976.33 ± 3.93 | 98.32 ± 0.04 | 1.05 ± 0.02 |
| 30% Ethanol | 25°C | - | 1920.77 ± 5.64 | 98.25 ± 0.05 | 1.00 ± 0.01 |
| | 50°C | - | 2009.21 ± 18.53 | 98.34 ± 0.01 | 1.03 ± 0.00 |
| | 75°C | - | 2159.55 ± 25.72 | 98.27 ± 0.11 | 0.96 ± 0.03 |
| 70% Ethanol | 25°C | - | 1566.66 ± 17.01 | 97.87 ± 0.06 | 0.78 ± 0.00 |
| | 50°C | - | 1819.21 ± 22.71 | 97.86 ± 0.03 | 0.90 ± 0.02 |
| | 75°C | - | 1954.33 ± 29.99 | 97.72 ± 0.09 | 0.92 ± 0.02 |
| 30% Methanol | 25°C | - | 1747.21 ± 22.96 | 98.22 ± 0.13 | 1.00 ± 0.01 |
| | 50°C | - | 1854.33 ± 25.22 | 98.32 ± 0.04 | 0.73 ± 0.01 |
| | 75°C | - | 2047.88 ± 26.00 | 98.27 ± 0.05 | 1.00 ± 0.01 |
| 70% Methanol | 25°C | - | 1829.88 ± 5.68 | 97.91 ± 0.32 | 0.95 ± 0.02 |
| | 50°C | - | 1842.77 ± 8.69$^{bf}$ | 98.12 ± 0.04$^{abcde}$ | 0.94 ± 0.03 |
| | 75°C | - | 2116.33 ± 24.34$^{eij}$ | 98.04 ± 0.02$^{abcde}$ | 0.97 ± 0.01 |

a-j Means ($n$ = 3)with different superscripts in a column of separate sample are significantly different ($p < 0.05$).

**Table S7.** Total anthocyanins and antioxidant capacity of dry - *Hylocereus* sp. under different extraction conditions.

| | | TAC (mg CGE/100g) | TPC (mg GAE/100g) | ABTS (%) | FRAP (mg/mL) |
|---|---|---|---|---|---|
| water | 25°C | - | 2030.55 ± 4.44 | 88.63 ± 0.49 | 0.93 ± 0.03 |
| | 50°C | - | 2311.21 ± 18.15 | 90.85 ± 0.17 | 0.76 ± 0.01 |
| | 75°C | - | 2229.10 ± 66.27 | 90.09 ± 0.88 | 0.67 ± 0.00 |
| 30% Ethanol | 25°C | - | 1759.33 ± 16.29 | 87.77 ± 0.27 | 0.85 ± 0.01 |
| | 50°C | - | 1885.44 ± 7.20 | 90.55 ± 0.06 | 0.92 ± 0.02 |
| | 75°C | - | 2044.88 ± 46.86 | 92.54 ± 0.41 | 0.78 ± 0.01 |
| 70% Ethanol | 25°C | - | 937.33 ± 7.33 | 77.80 ± 0.32 | 1.00 ± 0.00 |
| | 50°C | - | 1093.88 ± 24.85 | 79.60 ± 0.94 | 1.04 ± 0.01 |
| | 75°C | - | 1326.44 ± 5.52 | 81.65 ± 1.01 | 1.07 ± 0.03 |
| 30% Methanol | 25°C | - | 1926.21 ± 27.68 | 88.42 ± 0.69 | 0.74 ± 0.02 |
| | 50°C | - | 1733.10 ± 6.05 | 86.91 ± 0.23 | 0.82 ± 0.00 |
| | 75°C | - | 1766.10 ± 4.03 | 88.01 ± 0.54 | 0.73 ± 0.01 |
| 70% Methanol | 25°C | 7.79 ± 4.17 | 1293.77 ± 2.01 | 77.37 ± 0.38 | 0.99 ± 0.01 |
| | 50°C | - | 1336.44 ± 13.19 | 77.63 ± 0.18 | 0.99 ± 0.00 |
| | 75°C | 11.69 ± 4.09 | 1614.10 ± 13.02 | 83.10 ± 0.63 | 1.05 ± 0.01 |

a-i Means ($n$ =3) with different superscripts in a column of separate sample are significantly different ($p < 0.05$).

**Table S8.** Total anthocyanins and antioxidant capacity of dry - *Hylocereus* sp. petal under different extraction conditions.

| | | TAC (mg CGE/100g) | TPC (mg GAE/100g) | ABTS (%) | FRAP (mg/mL) |
|---|---|---|---|---|---|
| water | 25°C | - | 1618.99 ± 34.40 | 82.56 ± 0.28 | 0.88 ± 0.01 |
| | 50°C | - | 1794.77 ± 39.21 | 82.98 ± 0.74 | 0.81 ± 0.00 |
| | 75°C | - | 1284.33 ± 34.53 | 86.12 ± 10.57 | 0.59 ± 0.02 |
| 30% Ethanol | 25°C | - | 1568.99 ± 24.64 | 84.54 ± 0.33 | 0.96 ± 0.01 |
| | 50°C | - | 1711.88 ± 50.22 | 86.00 ± 1.90 | 0.91 ± 0.01 |
| | 75°C | - | 1542.44 ± 61.79 | 84.23 ± 0.50 | 0.70 ± 0.02 |
| 70% Ethanol | 25°C | - | 1054.88 ± 11.07 | 70.37 ± 1.09 | 0.79 ± 0.00 |
| | 50°C | - | 1176.77 ± 6.52 | 70.54 ± 0.76 | 0.83 ± 0.02 |
| | 75°C | - | 1411.55 ± 15.95 | 81.90 ± 14.25 | 0.80 ± 0.01 |
| 30% Methanol | 25°C | - | 1460.10 ± 18.34 | 77.99 ± 0.23 | 0.76 ± 0.00 |
| | 50°C | - | 1696.33 ± 25.11 | 82.33 ± 1.52 | 0.87 ± 0.02 |
| | 75°C | - | 1652.10 ± 14.22 | 82.13 ± 0.87 | 0.64 ± 0.01 |
| 70% Methanol | 25°C | 10.58 ± 3.44 | 1306.55 ± 5.52 | 71.32 ± 0.81 | 0.78 ± 0.02 |
| | 50°C | 6.68 ± 3.98 | 1401.44 ± 23.06 | 74.96 ± 2.40 | 0.87 ± 0.02 |
| | 75°C | - | 1570.55 ± 6.16 | 75.90 ± 0.87 | 0.83 ± 0.01 |

a-i Means ($n$ = 3) with different superscripts in a column of separate sample are significantly different ($p < 0.05$).

**Table S9.** Total anthocyanins and antioxidant capacity of dry - *Hylocereus* sp. Stamen under different extraction conditions.

| | | TAC (mg CGE/100g) | TPC (mg GAE/100g) | ABTS (%) | FRAP (mg/mL) |
|---|---|---|---|---|---|
| water | 25°C | - | 3406.44 ± 126.62 | 98.07 ± 0.30 | 0.89 ± 0.03 |
| | 50°C | - | 3358.99 ± 126.10 | 98.24 ± 0.02 | 0.90 ± 0.02 |
| | 75°C | - | 3055.99 ± 101.88 | 98.28 ± 0.04 | 0.71 ± 0.02 |
| 30% Ethanol | 25°C | - | 2570.10 ± 61.56 | 98.32 ± 0.01 | 0.88 ± 0.01 |
| | 50°C | - | 2824.21 ± 73.05 | 98.30 ± 0.01 | 0.87 ± 0.01 |
| | 75°C | - | 3117.44 ± 76.08 | 98.31 ± 0.01 | 0.82 ± 0.01 |
| 70% Ethanol | 25°C | - | 1816.21 ± 24.60 | 98.29 ± 0.32 | 0.96 ± 0.00 |
| | 50°C | - | 1820.55 ± 39.67 | 94.50 ± 0.32 | 0.92 ± 0.01 |
| | 75°C | - | 2117.66 ± 49.51 | 97.19 ± 0.61 | 0.98 ± 0.04 |
| 30% Methanol | 25°C | - | 2686.10 ± 62.80 | 98.32 ± 0.04 | 0.86 ± 0.01 |
| | 50°C | - | 2758.33 ± 66.11 | 98.33 ± 0.04 | 0.86 ± 0.01 |
| | 75°C | - | 2828.88 ± 72.97 | 98.27 ± 0.01 | 0.78 ± 0.01 |
| 70% Methanol | 25°C | 18.15 ± 1.30 | 2362.21 ± 46.54 | 97.99 ± 0.21 | 0.90 ± 0.01 |
| | 50°C | 16.70 ± 4.09 | 1813.44 ± 14.63 | 98.30 ± 0.03 | 0.87 ± 0.02 |
| | 75°C | - | 2093.66 ± 20.03 | 98.31 ± 0.03 | 0.93 ± 0.01 |

a-i Means ($n$ = 3) with different superscripts in a column of separate sample are significantly different ($p$ < 0.05).

**Table S10.** Total anthocyanins and antioxidant capacity of wet - *Hylocereus* sp. petal under different extraction conditions.

| | | TAC (mg CGE/100g) | TPC (mg GAE/100g) | ABTS (%) | FRAP (mg/mL) |
|---|---|---|---|---|---|
| water | 25°C | - | 58.88 ± 6.19 | 57.92 ± 1.09 | 0.08 ± 0.00 |
| | 50°C | - | 69.66 ± 2.73 | 56.66 ± 0.69 | 0.09 ± 0.00 |
| | 75°C | - | 127.55 ± 3.42 | 69.38 ± 6.13 | 0.14 ± 0.00 |
| 30% Ethanol | 25°C | - | 62.21 ± 4.60 | 59.05 ± 0.91 | 0.06 ± 0.00 |
| | 50°C | - | 66.66 ± 3.61 | 58.89 ± 0.68 | 0.04 ± 0.00 |
| | 75°C | - | 82.21 ± 3.610 | 59.56 ± 4.45 | 0.08 ± 0.00 |
| 70% Ethanol | 25°C | - | 56.10 ± 4.35 | 59.33 ± 1.45 | 0.04 ± 0.00 |
| | 50°C | - | 83.21 ± 4.60 | 65.50 ± 0.62 | 0.06 ± 0.01 |
| | 75°C | - | 105.66 ± 6.44 | 66.36 ± 0.62 | 0.06 ± 0.01 |
| 30% Methanol | 25°C | - | 54.77 ± 4.55 | 51.54 ± 0.23 | 0.05 ± 0.00 |
| | 50°C | - | 83.99 ± 1.86 | 54.42 ± 0.71 | 0.07 ± 0.00 |
| | 75°C | - | 85.88 ± 2.22 | 57.81 ± 3.23 | 0.07 ± 0.01 |
| 70% Methanol | 25°C | - | 60.88 ± 1.64 | 55.67 ± 0.52 | 0.05 ± 0.00 |
| | 50°C | - | 77.66 ± 2.60 | 61.06 ± 0.81 | 0.07 ± 0.00 |
| | 75°C | - | 85.44 ± 9.01 | 64.57 ± 0.74 | 0.08 ± 0.01 |

a-h Means ($n$ = 3) with different superscripts in a column of separate sample are significantly different ($p < 0.05$).

**Table S11.** Total anthocyanins and antioxidant capacity of wet - *Hylocereus* sp. Stamen under different extraction conditions.

| | | TAC (mg CGE/100g) | TPC (mg GAE/100g) | ABTS (%) | FRAP (mg/mL) |
|---|---|---|---|---|---|
| water | 25°C | - | 162.99 ± 4.26 | 81.36 ± 0.12 | 0.03 ± 0.00 |
| | 50°C | - | 181.99 ± 2.73 | 88.19 ± 0.27 | 0.03 ± 0.00 |
| | 75°C | - | 164.21 ± 2.14 | 83.59 ± 0.38 | 0.05 ± 0.00 |
| 30% Ethanol | 25°C | - | 115.33 ± 2.52 | 73.21 ± 0.46 | 0.02 ± 0.00 |
| | 50°C | - | 140.44 ± 3.86 | 82.77 ± 0.28 | 0.02 ± 0.00 |
| | 75°C | - | 177.88 ± 2.17 | 89.53 ± 0.59 | 0.10 ± 0.00 |
| 70% Ethanol | 25°C | - | 83.77 ± 1.17 | 72.80 ± 0.49 | 0.06 ± 0.00b |
| | 50°C | - | 201.21 ± 10.82 | 88.84 ± 0.78 | 0.06 ± 0.00 |
| | 75°C | - | 244.66 ± 5.24 | 90.30 ± 0.76 | 0.11 ± 0.01 |
| 30% Methanol | 25°C | - | 108.55 ± 6.74 | 77.09 ± 0.34 | 0.03 ± 0.00 |
| | 50°C | - | 155.77 ± 5.34 | 84.55 ± 0.17 | 0.01 ± 0.00 |
| | 75°C | - | 196.10 ± 4.86 | 87.27 ± 0.54 | 0.08 ± 0.00 |
| 70% Methanol | 25°C | - | 106.21 ± 6.05 | 70.74 ± 0.34 | 0.03 ± 0.00 |
| | 50°C | - | 138.99 ± 3.84 | 84.55 ± 0.76 | 0.06 ± 0.01 |
| | 75°C | - | 172.33 ± 1.33 | 89.11 ± 0.74 | 0.08 ± 0.00 |

a-i Means ($n$ = 3) with different superscripts in a column of separate sample are significantly different ($p$ < 0.05).

**Table S12.** Total anthocyanins and antioxidant capacity of *Dracaena* 1 under different extraction conditions.

| | | TAC (mg CGE/100g) | TPC (mg GAE/100g) | ABTS (%) | FRAP (mg/mL) |
|---|---|---|---|---|---|
| water | 25°C | - | 50.77 ± 7.19 | 1.54 ± 0.00 | - |
| | 50°C | - | 25.21 ± 3.67 | 0.38 ± 0.33 | - |
| | 75°C | - | 18.66 ± 3.21 | 1.30 ± 0.51 | - |
| 30% Ethanol | 25°C | - | 28.33 ± 1.00 | 14.71 ± 0.67 | 0.05 ± 0.00 |
| | 50°C | - | 73.33 ± 4.33 | 11.58 ± 0.87 | 0.04 ± 0.00 |
| | 75°C | - | 58.33 ± 1.76 | 12.53 ± 1.17 | 0.03 ± 0.00 |
| 70% Ethanol | 25°C | - | 145.44 ± 6.62 | 21.81 ± 1.78 | 0.15 ± 0.00 |
| | 50°C | 7.12 ± 28.47 | 150.99 ± 2.03 | 20.95 ± 1.62 | 0.15 ± 0.00 |
| | 75°C | - | 145.33 ± 3.21 | 19.58 ± 0.84 | 0.14 ± 0.01 |
| 30% Methanol | 25°C | - | 33.55 ± 4.07 | 5.02 ± 0.38 | 0.03 ± 0.00 |
| | 50°C | - | 46.33 ± 1.67 | 3.49 ± 0.17 | 0.02 ± 0.00 |
| | 75°C | - | 36.10 ± 4.86 | 5.58 ± 0.43 | 0.01 ± 0.00 |
| 70% Methanol | 25°C | - | 61.21 ± 2.12 | 13.32 ± 0.29 | 0.06 ± 0.00 |
| | 50°C | - | 82.33 ± 0.88 | 15.22 ± 1.05 | 0.07 ± 0.01 |
| | 75°C | - | 86.44 ± 2.55 | 15.65 ± 1.06 | 0.07 ± 0.00 |

a-h Means ($n$ = 3) with different superscripts in a column of separate sample are significantly different ($p < 0.05$).

**Table S13.** Total anthocyanins and antioxidant capacity of *Dracaena* 2 under different extraction conditions.

| | | TAC (mg CGE/100g) | TPC (mg GAE/100g) | ABTS (%) | FRAP (mg/mL) |
|---|---|---|---|---|---|
| water | 25°C | - | 4.33 ± 6.03 | 41.94 ± 7.43 | - |
| | 50°C | - | 5.10 ± 4.54 | 38.68 ± 0.67 | - |
| | 75°C | - | 8.10 ± 2.83 | 40.42 ± 0.59 | 0.00 ± 0.00 |
| 30% Ethanol | 25°C | - | 23.66 ± 4.06 | 45.31 ± 0.33 | 0.03 ± 0.00 |
| | 50°C | - | 27.44 ± 1.84 | 46.25 ± 7.28 | 0.04 ± 0.00 |
| | 75°C | - | 55.55 ± 1.64 | 59.07 ± 0.38 | 0.08 ± 0.00 |
| 70% Ethanol | 25°C | - | 77.88 ± 2.17 | 64.38 ± 0.53 | 0.13 ± 0.00 |
| | 50°C | - | 125.88 ± 6.16 | 71.10 ± 0.60 | 0.16 ± 0.01 |
| | 75°C | - | 149.33 ± 2.03 | 75.07 ± 1.34 | 0.18 ± 0.01 |
| 30% Methanol | 25°C | - | 30.99 ± 4.73 | 40.70 ± 0.38 | 0.02 ± 0.00 |
| | 50°C | - | 42.10 ± 1.02 | 45.48 ± 0.07 | 0.03 ± 0.00 |
| | 75°C | - | 61.33 ± 1.00 | 49.11 ± 0.41 | 0.04 ± 0.00 |
| 70% Methanol | 25°C | - | 77.55 ± 3.66 | 51.81 ± 0.45 | 0.06 ± 0.00 |
| | 50°C | - | 21.21 ± 5.87 | 57.47 ± 0.70 | 0.9 ± 0.00 |
| | 75°C | - | 26.88 ± 2.22 | 63.08 ± 0.25 | 0.13 ± 0.00 |

a-j Means ($n$ = 3) with different superscripts in a column of separate sample are significantly different ($p < 0.05$).

**Table S14.** Total anthocyanins and antioxidant capacity of *Dracaena* 3 under different extraction conditions.

| | | TAC (mg CGE/100g) | TPC (mg GAE/100g) | ABTS (%) | FRAP (mg/mL) |
|---|---|---|---|---|---|
| water | 25°C | - | 0.10 ± 3.72 | 37.89 ± 0.66 | - |
| | 50°C | - | 1.77 ± 2.67 | 38.29 ± 0.06 | - |
| | 75°C | - | 2.66 ± 6.65 | 40.64 ± 0.59 | - |
| 30% Ethanol | 25°C | - | 19.33 ± 2.40 | 47.07 ± 0.27 | 0.01 ± 0.00 |
| | 50°C | - | 23.88 ± 3.56 | 54.05 ± 0.08 | 0.03 ± 0.00 |
| | 75°C | - | 37.44 ± 2.41 | 63.55 ± 0.89 | 0.04 ± 0.00 |
| 70% Ethanol | 25°C | - | 23.88 ± 3.56 | 74.07 ± 0.81 | 0.11 ± 0.00 |
| | 50°C | - | 37.44 ± 2.41 | 82.92 ± 2.03 | 0.15 ± 0.00 |
| | 75°C | - | 120.88 ± 2.83 | 82.78 ± 2.65 | 0.18 ± 0.01 |
| 30% Methanol | 25°C | - | 30.33 ± 1.20 | 41.01 ± 3.14 | - |
| | 50°C | - | 70.21 ± 2.50 | 43.28 ± 0.55 | 0.00 ± 0.00 |
| | 75°C | - | 72.66 ± 0.33 | 49.62 ± 1.21 | 0.02 ± 0.00 |
| 70% Methanol | 25°C | - | 11.44 ± 6.50 | 57.73 ± 0.87 | 0.06 ± 0.00 |
| | 50°C | - | 8.21 ± 8.98 | 69.94 ± 0.17 | 0.11 ± 0.01 |
| | 75°C | - | 21.10 ± 4.55 | 70.17 ± 2.02 | 0.12 ± 0.00 |

a-g Means ($n$ = 3) with different superscripts in a column of separate sample are significantly different ($p < 0.05$).

**Table S15.** Total anthocyanins and antioxidant capacity of *Dracaena* 4 under different extraction conditions.

| | | TAC (mg CGE/100g) | TPC (mg GAE/100g) | ABTS (%) | FRAP (mg/mL) |
|---|---|---|---|---|---|
| water | 25°C | - | 0.33 ± 6.17 | 0.78 ± 0.66 | - |
| | 50°C | - | 129.21 ± 0.51 | 1.23 ± 0.39 | - |
| | 75°C | - | 18.33 ±3.18 | 6.52 ± 2.02 | 0.00 ± 0.00 |
| 30% Ethanol | 25°C | - | 113.77 ± 2.52 | 11.59 ± 1.51 | 0.04 ± 0.00 |
| | 50°C | 1.12 ± 0.04 | 151.77 ± 0.51 | 18.21 ± 2.72 | 0.08 ± 0.00 |
| | 75°C | 0.08 ± 0.08 | 176.77 ± 4.95 | 23.07 ± 1.52 | 0.11 ± 0.00 |
| 70% Ethanol | 25°C | 2.24 ± 17.49 | 449.100 ± 14.91 | 31.45 ± 0.24 | 0.22 ± 0.01 |
| | 50°C | 21.24 ± 47.54 | 507.44 ± 9.41 | 31.41 ± 0.31 | 0.22 ± 0.01 |
| | 75°C | - | 537.88 ± 10.40 | 33.97 ± 0.29 | 0.23 ± 0.02 |
| 30% Methanol | 25°C | - | 20.66 ± 7.80 | 3.60 ± 0.95 | - |
| | 50°C | - | 46.21 ± 4.60 | 6.36 ± 0.24 | 0.01 ± 0.00 |
| | 75°C | - | 92.66 ± 6.17 | 13.27 ± 0.70 | 0.05 ± 0.00 |
| 70% Methanol | 25°C | 4.57 ± 2.74 | 266.99 ± 2.73 | 23.12 ± 0.34 | 0.16 ± 0.01 |
| | 50°C | 8.04 ± 8.34 | 413.55 ± 12.54 | 27.74 ± 0.66 | 0.19 ± 0.01 |
| | 75°C | 41.74 ± 17.05 | 486.77 ± 11.61 | 28.51 ± 0.94 | 0.25 ± 0.01 |

a-j Means ($n = 3$) with different superscripts in a column of separate sample are significantly different ($p < 0.05$).

**Table S16.** Total anthocyanins and antioxidant capacity of *Dracaena* 5 under different extraction conditions.

| | | TAC (mg CGE/100g) | TPC (mg GAE/100g) | ABTS (%) | FRAP (mg/mL) |
|---|---|---|---|---|---|
| water | 25°C | - | 8.66 ± 3.51 | 1.73 ± 0.66 | - |
| | 50°C | - | 6.88 ± 5.59 | 0.23 ± 0.14 | - |
| | 75°C | - | 46.44 ± 4.50 | 3.19 ± 1.49 | - |
| 30% Ethanol | 25°C | - | 15.33 ± 1.15 | 9.54 ± 0.13 | 0.01 ± 0.00 |
| | 50°C | - | 83.55 ± 4.44 | 12.99 ± 1.54 | 0.03 ± 0.00 |
| | 75°C | - | 34.21 ± 3.15 | 17.43 ± 0.84 | 0.02 ± 0.00 |
| 70% Ethanol | 25°C | 9.31 ± 15.46 | 125.99 ± 2.08 | 29.46 ± 0.98 | 0.12 ± 0.00 |
| | 50°C | - | 69.88 ± 3.34 | 22.40 ± 1.98 | 0.08 ± 0.00 |
| | 75°C | - | 120.10 ± 1.68 | 33.20 ± 0.73 | 0.14 ± 0.01 |
| 30% Methanol | 25°C | - | 22.44 ± 6.19 | 3.08 ± 0.13 | 0.00 ± 0.00 |
| | 50°C | - | 22.44 ± 4.44 | 9.12 ± 0.71 | 0.01 ± 0.00 |
| | 75°C | - | 24.21 ± 5.19 | 12.53 ± 0.46 | 0.02 ± 0.00 |
| 70% Methanol | 25°C | - | 54.66 ± 3.51 | 18.37 ± 0.98 | 0.06 ± 0.00 |
| | 50°C | - | 72.33 ± 1.76 | 24.86 ± 0.77 | 0.08 ± 0.01 |
| | 75°C | - | 121.99 ± 1.20 | 28.57 ± 0.88 | 0.11 ± 0.00 |

a-i Means ($n = 3$) with different superscripts in a column of separate sample are significantly different ($p < 0.05$).

**Table S17.** Total anthocyanins and antioxidant capacity of *Dracaena* 6 under different extraction conditions.

| | | TAC (mg CGE/100g) | TPC (mg GAE/100g) | ABTS (%) | FRAP (mg/mL) |
|---|---|---|---|---|---|
| water | 25°C | - | 13.33 ± 6.08 | 2.02 ± 0.00 | - |
| | 50°C | - | 4.88 ± 2.22 | 1.19 ± 0.00 | - |
| | 75°C | - | 7.44 ± 2.41 | 5.11 ± 0.42 | - |
| 30% Ethanol | 25°C | - | 17.99 ± 2.00 | 9.92 ± 0.65 | 0.01 ± 0.00 |
| | 50°C | - | 29.21 ± 2.04 | 14.43 ± 1.08 | 0.03 ± 0.00 |
| | 75°C | 0.09 ± 0.11 | 26.88 ± 2.01 | 15.75 ± 0.50 | 0.03 ± 0.00 |
| 70% Ethanol | 25°C | 0.67 ± 6.22 | 96.21 ± 0.77 | 26.06 ± 0.23 | 0.11 ± 0.00 |
| | 50°C | 2.08 ± 3.08 | 92.77 ± 2.34 | 22.92 ± 1.69 | 0.09 ± 0.00 |
| | 75°C | 3.25 ± 4.53 | 119.55 ± 3.15 | 26.29 ±0.35 | 0.09 ± 0.01 |
| 30% Methanol | 25°C | 0.11 ± 0.11 | 17.66 ± 4.58 | 5.55 ± 0.74 | - |
| | 50°C | - | 20.88 ± 1.84 | 11.08 ± 0.37 | 0.01 ± 0.00 |
| | 75°C | - | 30.21 ± 7.71 | 11.24 ± 0.74 | 0.01 ± 0.00 |
| 70% Methanol | 25°C | - | 59.10 ± 0.38 | 19.03 ± 0.21 | 0.06 ± 0.00 |
| | 50°C | 2.97 ± 5.54 | 74.21 ± 2.01 | 21.97 ± 0.68 | 0.08 ± 0.00 |
| | 75°C | 1.14 ± 1.54 | 65.21 ± 1.64 | 18.41 ± 0.4 | 0.06 ± 0.00 |

a-j Means ($n$ = 3) with different superscripts in a column of separate sample are significantly different ($p$ < 0.05).

**Table S18.** Total anthocyanins and antioxidant capacity of *Dracaena* 7 under different extraction conditions.

| | | TAC (mg CGE/100g) | TPC (mg GAE/100g) | ABTS (%) | FRAP (mg/mL) |
|---|---|---|---|---|---|
| water | 25°C | - | - | - | - |
| | 50°C | - | - | - | - |
| | 75°C | - | 3.55 ± 1.26 | 2.28 ± 0.00 | - |
| 30% Ethanol | 25°C | - | 24.21 ± 2.01 | 3.95 ± 1.36 | - |
| | 50°C | - | 31.33 ± 4.91 | 10.41 ± 0.91 | 0.02 ± 0.00 |
| | 75°C | - | 34.44 ± 0.84 | 22.40 ± 2.15 | 0.05 ± 0.00 |
| 70% Ethanol | 25°C | - | 135.66 ± 0.58 | 33.35 ± 1.64 | 0.12 ± 0.00 |
| | 50°C | 7.98 ± 8.53 | 144.55 ± 2.17 | 39.99 ± 1.66 | 0.16 ± 0.00 |
| | 75°C | - | 214.77 ± 3.75 | 43.92 ± 0.83 | 0.19 ± 0.01 |
| 30% Methanol | 25°C | - | 10.33 ± 5.51 | 0.13 ± 0.00 | - |
| | 50°C | - | 12.33 ± 4.04 | 4.70 ± 0.23 | 0.00 ± 0.00 |
| | 75°C | - | 28.77 ± 1.35 | 7.40 ± 0.53 | 0.02 ± 0.00 |
| 70% Methanol | 25°C | - | 71.55 ± 3.91 | 20.85 ± 1.55 | 0.06 ± 0.00 |
| | 50°C | - | 97.33 ± 1.73 | 30.97 ± 1.33 | 0.11 ± 0.01 |
| | 75°C | - | 209.66 ± 1.45 | 44.81 ± 0.83 | 0.18 ± 0.00 |

a-g Means ($n$ = 3) with different superscripts in a column of separate sample are significantly different ($p$ < 0.05).

**Table S19.** Total anthocyanins and antioxidant capacity of *Dracaena* 8 under different extraction conditions.

| | | TAC (mg CGE/100g) | TPC (mg GAE/100g) | ABTS (%) | FRAP (mg/mL) (mg/mL) |
|---|---|---|---|---|---|
| water | 25°C | - | 2.88 ± 5.01 | 16.58 ± 0.88 | - |
| | 50°C | - | 4.77 ± 4.40 | 18.09 ± 0.21 | - |
| | 75°C | - | 6.77 ± 7.76 | 19.94 ± 0.95 | 0.00 ± 0.00 |
| 30% Ethanol | 25°C | - | 23.44 ± 0.84 | 32.94 ± 0.67 | 0.01 ± 0.00 |
| | 50°C | - | 35.88 ± 6.62 | 26.28 ± 0.69 | 0.02 ± 0.00 |
| | 75°C | - | 63.99 ± 4.04 | 52.59 ± 1.14 | 0.07 ± 0.00 |
| 70% Ethanol | 25°C | - | 88.44 ± 2.83 | 58.33 ± 1.38 | 0.09 ± 0.00 |
| | 50°C | - | 125.10 ± 3.02 | 72.68 ± 0.75 | 0.14 ± 0.00 |
| | 75°C | - | 183.66 ± 2.85 | 80.00 ± 0.63 | 0.18 ± 0.01 |
| 30% Methanol | 25°C | - | 14.88 ± 5.42 | 22.48 ± 0.69 | - |
| | 50°C | - | 25.10 ± 4.67 | 27.04 ± 0.23 | 0.01 ± 0.00 |
| | 75°C | - | 32.88 ± 3.40 | 31.95 ± 0.93 | 0.05 ± 0.00 |
| 70% Methanol | 25°C | - | 48.33 ± 2.85 | 43.62 ± 0.35 | 0.03 ± 0.00 |
| | 50°C | - | 91.44 ± 7.72 | 60.79 ± 0.13 | 0.08 ± 0.00 |
| | 75°C | - | 119.66 ± 0.67 | 66.27 ± 2.20 | 0.10 ± 0.00 |

a-k Means ($n = 3$) with different superscripts in a column of separate sample are significantly different ($p < 0.05$).

**Table S20.** Total anthocyanins and antioxidant capacity of *Dracaena* 9 under different extraction conditions.

| | | TAC (mg CGE/100g) | TPC (mg GAE/100g) | ABTS (%) | FRAP (mg/mL) |
|---|---|---|---|---|---|
| water | 25°C | - | 125.55 ± 4.67 | 17.75 ± 0.21 | 0.05 ± 0.00 |
| | 50°C | - | 254.66 ± 2.19 | 31.40 ± 0.40 | 0.20 ± 0.00 |
| | 75°C | - | 226.10 ± 1.07 | 35.78 ± 1.76 | 0.23 ± 0.00 |
| 30% Ethanol | 25°C | - | 311.66 ± 1.86 | 50.06 ± 1.33 | 0.41 ± 0.00 |
| | 50°C | - | 548.21 ± 3.75 | 76.25 ± 1.91 | 0.63 ± 0.00 |
| | 75°C | - | 649.33 ± 16.56 | 85.58 ± 1.60 | 0.67 ± 0.01 |
| 70% Ethanol | 25°C | - | 710.77 ± 8.92 | 89.96 ± 1.20 | 1.34 ± 0.01 |
| | 50°C | - | 544.21 ± 2.52 | 81.12 ± 1.23 | 0.98 ± 0.01 |
| | 75°C | - | 446.44 ± 6.26 | 72.16 ± 1.31 | 1.46 ± 0.03 |
| 30% Methanol | 25°C | - | 317.99 ± 3.71 | 42.61 ± 1.74 | 0.22 ± 0.00 |
| | 50°C | - | 445.55 ± 7.07 | 58.59 ± 1.26 | 0.46 ± 0.00 |
| | 75°C | - | 416.55 ± 3.50 | 60.50 ± 0.90 | 0.46 ± 0.00 |
| 70% Methanol | 25°C | - | 651.44 ± 0.84 | 87.84 ± 0.64 | 1.26 ± 0.03 |
| | 50°C | - | 936.88 ± 6.48 | 91.99 ± 1.47 | 1.42 ± 0.01 |
| | 75°C | - | 734.44 ± 4.74 | 86.17 ± 1.67 | 1.34 ± 0.01 |

a-l Means ($n$ = 3) with different superscripts in a column of separate sample are significantly different ($p$ < 0.05).

**Table S21.** Total anthocyanins and antioxidant capacity of *Dracaena cinnabari* under different extraction conditions.

| | | TAC (mg CGE/100g) | TPC (mg GAE/100g) | ABTS (%) | FRAP (mg/mL) |
|---|---|---|---|---|---|
| water | 25°C | - | 53.88 ± 4.03 | 11.04 ± 0.77 | 0.10 ± 0.00 |
| | 50°C | - | 93.55 ± 4.68 | 23.32 ± 1.13 | 0.20 ± 0.00 |
| | 75°C | - | 128.10 ± 6.34 | 30.82 ± 0.26 | 0.22 ± 0.00 |
| 30% Ethanol | 25°C | - | 440.33 ± 2.40 | 72.43 ± 1.28 | 0.32 ± 0.00 |
| | 50°C | - | 474.33 ± 3.93 | 72.83 ± 0.10 | 0.30 ± 0.00 |
| | 75°C | - | 768.88 ± 1.64 | 85.05 ± 0.82 | 0.65 ± 0.01 |
| 70% Ethanol | 25°C | - | 1249.33 ± 4.91 | 93.93 ± 0.81 | 2.17 ± 0.03 |
| | 50°C | 16.76 ± 17.7 | 1257.66 ± 13.72 | 92.85 ± 0.93 | 2.08 ± 0.05 |
| | 75°C | - | 1648.33 ± 25.00 | 94.59 ± 0.09 | 2.35 ± 0.04 |
| 30% Methanol | 25°C | - | 218.55 ± 1.26 | 41.66 ± 1.47 | 0.20 ± 0.00 |
| | 50°C | - | 368.10 ± 4.86 | 63.17 ± 2.17 | 0.25 ± 0.00 |
| | 75°C | - | 517.44 ± 7.50 | 78.57 ± 0.44 | 0.39 ± 0.01 |
| 70% Methanol | 25°C | 104.97 ± 23.37 | 1372.66 ± 14.19 | 93.93 ± 0.14 | 1.74 ± 0.05 |
| | 50°C | 49.88 ± 11.08 | 1100.44 ± 27.57 | 91.17 ± 0.60 | 1.50 ± 0.02 |
| | 75°C | 89.35 ± 22.10 | 1413.44 ± 10.40 | 94.21 ± 0.44 | 1.99 ± 0.02 |

a-m Means ($n$ = 3) with different superscripts in a column of separate sample are significantly different ($p < 0.05$).

**Table S22.** Total anthocyanins and antioxidant capacity of *Dracaena cochinchinensis* under different extraction conditions.

| | | TAC (mg CGE/100g) | TPC (mg GAE/100g) | ABTS (%) | FRAP (mg/mL) |
|---|---|---|---|---|---|
| water | 25°C | - | 219.21 ± 8.57 | 88.47 ± 0.29 | 0.12 ± 0.00 |
| | 50°C | - | 288.77 ± 3.86 | 91.55 ± 0.09 | 0.19 ± 0.00 |
| | 75°C | - | 316.66 ± 1.45 | 92.20 ± 0.31 | 0.20 ± 0.00 |
| 30% Ethanol | 25°C | - | 905.88 ± 13.67 | 98.11 ± 0.01 | 0.26 ± 0.00 |
| | 50°C | - | 927.55 ± 15.35 | 97.81 ± 0.05 | 0.69 ± 0.00 |
| | 75°C | - | 2079.99 ± 11.57 | 98.19 ± 0.04 | 0.47 ± 0.01 |
| 70% Ethanol | 25°C | - | 3183.88 ± 33.49 | 97.68 ± 0.06 | 0.60 ± 0.01 |
| | 50°C | 6.57 ± 2.85 | 3712.77 ± 31.29 | 97.44 ± 0.20 | 0.46 ± 0.01 |
| | 75°C | - | 3677.44 ± 59.16 | 97.42 ± 0.04 | 0.38 ± 0.01 |
| 30% Methanol | 25°C | - | 544.99 ± 7.13 | 97.54 ± 0.08 | 0.23 ± 0.00 |
| | 50°C | - | 571.99 ± 13.69 | 98.03 ± 0.17 | 0.35 ± 0.01 |
| | 75°C | - | 1089.21 ± 4.50 | 98.31 ± 0.03 | 0.34 ± 0.01 |
| 70% Methanol | 25°C | 1.93 ± 5.43 | 4068.55 ± 78.09 | 96.13 ± 0.23 | 0.58 ± 0.00 |
| | 50°C | 5.04 ± 3.14 | 4715.21 ± 73.58 | 95.34 ± 0.21 | 0.90 ± 0.02 |
| | 75°C | 55.34 ± 23.49 | 3442.33 ± 102.09 | 96.33 ± 0.38 | 0.62 ± 0.00 |

a-k Means ($n$ = 3) with different superscripts in a column of separate sample are significantly different ($p$ < 0.05).

**Table S23.** Total anthocyanins and antioxidant capacity of *Daemonorops draco* under different extraction conditions.

| | | TAC (mg CGE/100g) | TPC (mg GAE/100g) | ABTS (%) | FRAP (mg/mL) |
|---|---|---|---|---|---|
| water | 25°C | - | 151.66 ± 4.18 | 22.72 ± 1.26 | 0.12 ± 0.01 |
| | 50°C | - | 943.77 ± 7.86 | 83.68 ± 0.61 | 0.70 ± 0.00 |
| | 75°C | - | 964.88 ± 7.12 | 82.92 ± 0.55 | 0.54 ± 0.01 |
| 30% Ethanol | 25°C | - | 876.21 ± 1.84 | 78.27 ± 1.49 | 0.54 ± 0.00 |
| | 50°C | - | 2088.55 ± 12.38 | 96.94 ± 0.06 | 1.30 ± 0.00 |
| | 75°C | - | 1882.33 ± 15.65 | 95.14 ± 0.50 | 1.07 ± 0.01 |
| 70% Ethanol | 25°C | - | 1133.44 ± 1.35 | 87.15 ± 1.55 | 0.71 ± 0.01 |
| | 50°C | - | 741.33 ± 6.23 | 72.46 ± 1.14 | 0.48 ± 0.01 |
| | 75°C | - | 1459.55 ± 10.30 | 90.06 ± 0.87 | 0.86 ± 0.03 |
| 30% Methanol | 25°C | - | 1327.33 ± 4.73 | 90.34 ± 0.28 | 0.82 ± 0.01 |
| | 50°C | - | 834.44 ± 5.98 | 72.55 ± 1.06 | 0.49 ± 0.00 |
| | 75°C | - | 806.21 ± 6.59 | 74.88 ± 0.86 | 0.55 ± 0.01 |
| 70% Methanol | 25°C | - | 417.99 ± 6.36 | 44.47 ± 2.38 | 0.32 ± 0.01 |
| | 50°C | - | 712.10 ± 2.50 | 68.04 ± 1.51 | 0.46 ± 0.01 |
| | 75°C | - | 637.77 ± 9.38 | 61.79 ± 0.34 | 0.45 ± 0.00 |

a-n Means ($n$ = 3) with different superscripts in a column of separate sample are significantly different ($p < 0.05$).

**Table S24.** Total Anthocyanins and Antioxidant Ability of Hybrid Natural Plants Using Optimum Mixtures.

| | **TAC (mg CGE/100g)** | **TPC (mg GAE/100g)** | **ABTS (%)** | **FRAP (mg/mL)** |
|---|---|---|---|---|
| CS+PA | 117.78 ± 5.43 | 9939.10 ± 81.25 | 92.73 ± 0.15 | 3.74 ± 0.06 |
| CS+H1 | 38.80 ± 2.60 | 10365.33 ± 263.59 | 88.83 ± 0.72 | 3.75 ± 0.03 |
| HS+CT+PA | 1765.41 ± 27.17 | 3700.88 ± 263.59 | 90.44 ± 0.31 | 2.32 ± 0.01 |
| CT+CS+PA | 216.92 ± 18.86 | 7952.55 ± 22.56 | 92.32 ± 0.22 | 3.69 ± 0.07 |
| CS+PA+H3 | 39.85 ± 3.32 | 7908.77 ± 92.58 | 88.11 ± 0.13 | 3.49 ± 0.07 |
| HS+CS+PA+H3 | 1256.65 ± 14.64 | 7316.99 ± 53.00 | 88.81 ± 0.18 | 3.60 ± 0.14 |
| CT+CS+PA+H1 | 131.59 ± 2.05 | 6808.99 ± 165.86 | 89.62 ± 0.17 | 3.51 ± 0.09 |
| HS+CT+CS+PA+H3 | 994.20 ± 40.09 | 6542.33 ± 76.98 | 88.17 ± 0.21 | 3.63 ± 0.02 |
| CS+DD | 15.28 ± 3.80 | 9509.77 ± 58.31 | 93.10 ± 0.34 | 3.71 ± 0.03 |
| CS+PA+DD | 53.48 ± 1.06 | 7541.10 ± 43.49 | 93.08 ± 0.09 | 3.64 ± 0.19 |
| CS+H1+DD | - | 7937.33 ± 47.33 | 90.86 ± 0.12 | 3.59 ± 0.07 |
| HS+CT+PA+DD | 965.15 ± 14.22 | 3113.88 ± 77.18 | 90.75 ± 0.46 | 1.86 ± 0.04 |
| CT+CS+PA+DD | 80.45 ± 7.94 | 6535.10 ± 176.63 | 92.61 ± 0.45 | 3.54 ± 0.08 |
| CS+PA+H3+DD | 19.79 ± 1.44 | 6439.66 ± 22.44 | 89.85 ± 0.26 | 3.54 ± 0.06 |
| HS+CS+PA+H3+DD | 791.39 ± 12.42 | 5892.99 ± 42.34 | 90.06 ± 0.17 | 3.61 ± 0.19 |
| CT+CS+PA+H1+DD | 77.57 ± 2.20 | 5907.21 ± 11.55 | 90.60 ± 0.45 | 3.47 ± 0.12 |
| HS+CT+CS+PA+H3+DD | 668.24 ± 13.75 | 5720.77 ± 60.64 | 89.60 ± 0.29 | 3.47 ± 0.03 |
| CS+DC | - | 9480.66 ± 179.61 | 92.50 ± 0.15 | 3.71 ± 0.07 |
| CS+PA+DC | 56.83 ± 26.50 | 8513.21 ± 51.81 | 90.73 ± 0.22 | 3.71 ± 0.08 |
| CS+H1+DC | 22.50 ± 9.07 | 7590.33 ± 63.79 | 90.95 ± 0.14 | 3.69 ± 0.01 |
| HS+CT+PA+DC | 940.00 ± 35.97 | 3920.66 ± 51.55 | 88.21 ± 0.64 | 1.83 ± 0.03 |
| CT+CS+PA+DC | 107.37 ± 44.29 | 7382.99 ± 160.06 | 90.13 ± 0.12 | 3.58 ± 0.03 |
| CS+PA+H3+DC | 37.57 ± 16.15 | 7028.21 ± 160.06 | 88.04 ± 0.46 | 3.66 ± 0.08 |
| HS+CS+PA+H3+DC | 851.91 ± 19.76 | 6212.44 ± 43.49 | 86.75 ± 0.25 | 3.54 ± 0.09 |
| CT+CS+PA+H1+DC | 120.59 ± 27.32 | 6537.99 ± 51.62 | 87.93 ± 0.25 | 3.44 ± 0.13 |
| HS+CT+CS+PA+H3+DC | 758.01 ± 15.83 | 6272.21 ± 268.32 | 87.46 ± 0.53 | 3.51 ± 0.04 |

a-g Means ($n$ = 3) with different superscripts in a column of separate sample are significantly different ($p < 0.05$).
